# Scaffolding Biomaterials for 3D Cultivated Meat: Prospects and Challenges

**DOI:** 10.1002/advs.202102908

**Published:** 2021-11-16

**Authors:** Claire Bomkamp, Stacey C. Skaalure, Gonçalo F. Fernando, Tom Ben‐Arye, Elliot W. Swartz, Elizabeth A. Specht

**Affiliations:** ^1^ The Good Food Institute 1380 Monroe St. NW #229 Washington DC 20010 USA

**Keywords:** biomaterials, bioprinting, cell‐based meat, cultivated meat, cultured meat, scaffolding, tissue engineering

## Abstract

Cultivating meat from stem cells rather than by raising animals is a promising solution to concerns about the negative externalities of meat production. For cultivated meat to fully mimic conventional meat's organoleptic and nutritional properties, innovations in scaffolding technology are required. Many scaffolding technologies are already developed for use in biomedical tissue engineering. However, cultivated meat production comes with a unique set of constraints related to the scale and cost of production as well as the necessary attributes of the final product, such as texture and food safety. This review discusses the properties of vertebrate skeletal muscle that will need to be replicated in a successful product and the current state of scaffolding innovation within the cultivated meat industry, highlighting promising scaffold materials and techniques that can be applied to cultivated meat development. Recommendations are provided for future research into scaffolds capable of supporting the growth of high‐quality meat while minimizing production costs. Although the development of appropriate scaffolds for cultivated meat is challenging, it is also tractable and provides novel opportunities to customize meat properties.

## Graphical abstract

Cultivating meat from cells is a promising solution to the environmental, ethical, health, and food‐security challenges associated with conventional meat production. Cultivated meat will require the development of scalable, low‐cost, and edible or biodegradable scaffolds to support cell growth. This review discusses the unique challenges of cultivated meat scaffolding and highlights promising materials and processing methods worthy of further investigation.

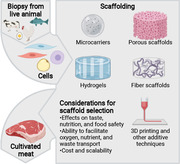

## 1 Introduction

Large‐scale conventional animal agriculture is associated with a host of environmental and public health issues. Primarily due to the increase in close human–animal contacts from animal agriculture, destruction of wildlife habitats, and rising human population and global mobility, 75% of new infectious diseases in humans arise from animal sources (zoonotic).^[^ ^1^ ^]^ Intensive animal farming also plays a substantial role in antibiotic resistance, as 80% of all antibiotics sold in the United States^[^ ^2^ ^]^ and 73% of antibiotics sold globally^[^ ^3^ ^]^ are administered to livestock. Much of the deforestation of sensitive habitats such as the Amazon rainforest, which irreversibly threatens biodiversity and brings humans close to displaced wild animals, is due to livestock grazing or feed cropping.^[^ ^4^ ^]^ A 2020 report from the UN Environment Programme on preventing future pandemics noted that two of the seven major anthropogenic causes of zoonotic disease are increased global demand for animal protein products and unsustainable agriculture intensification, such as the rise of intensive animal agriculture.^[^ ^5^ ^]^

When assessed according to CO_2_ equivalents, greenhouse gas emissions from livestock represent 14.5% of anthropogenic emissions.^[^ ^6^ ^]^ In addition to climate impacts, meat production comes at a high environmental cost in areas such as land and water use, primarily due to animal feed production and animal waste contamination.^[^ ^7^ ^]^ Public concerns around climate change appear to drive purchasing behavior at least to some extent, as increased media coverage of climate change is associated with decreased demand for beef.^[^ ^8^ ^]^ However, global meat consumption continues to rise steadily^[^ ^7^, ^9^ ^]^ despite increased public awareness of climate change, suggesting that the desire to avoid climate impacts is, by itself, not sufficient to meaningfully curb meat consumption.

A possibly more pragmatic means of addressing the negative externalities of meat production is by changing the production process rather than requiring large‐scale consumer behavior change. Cultivated meat (CM; also called cell‐based or cultured meat) is meat grown from animal stem cells, mimicking the process by which cells grow and divide in vivo to produce a product with the same nutritional and organoleptic properties as its conventional counterpart.^[^ ^10^, ^11^, ^12^, ^13^, ^14^ ^]^ The first experimental demonstration of CM was reported in 2002, which showed that cultured fish cells could contribute to the growth of a goldfish muscle explant.^[^ ^15^ ^]^ The first reported tasting of CM occurred in 2013 with the much‐publicized hamburger produced by Dr. Mark Post's team.^[^ ^16^ ^]^ Today, a growing number of companies (at least 70 as of mid‐2021) are working to commercialize and scale CM.^[^ ^17^ ^]^ The first regulatory approval for cultivated chicken occurred in December 2020 in Singapore, with commercial sales following shortly thereafter.^[^ ^18^ ^]^

Two recent reports informed by empirical data from 15 companies involved in the CM supply chain model the possible costs and environmental impacts of commercial‐scale CM production. The life cycle assessment (LCA) found that CM could vastly outperform conventional meat with regard to resource utilization. At the same time, its climate impacts are highly dependent on the energy source used.^[^ ^19^ ^]^ In addition, the LCA found that CM could reduce land use by 63–95%. Still, it does not account for the potential for carbon capture by the newly available land,^[^ ^20^ ^]^ e.g., by rewilding and ecosystem restoration.^[^ ^21^ ^]^ CM is not a “silver bullet” for climate concerns, but its potential synergies with other approaches such as clean energy and rewilding offer a promising opportunity to substantially reduce the climate impacts of our food system. The accompanying techno‐economic assessment (TEA) found that a commercial‐scale CM production process could compete on costs with some forms of conventional meat. However, further improvements must be made to reduce the costs of growth factors and proteins, develop better methods for high‐density cell culture, and create more efficient bioreactors.^[^ ^22^ ^]^

A critical step in many existing and hypothetical bioprocesses is the seeding of cells on a 3D scaffold.^[^ ^23^ ^]^ The scaffold often plays a vital role in ensuring the efficient transport of oxygen, nutrients, and waste products to and from the cells, controlling the growing tissue's geometry and cell type distribution, and contributing structure to the final product. In addition, cells have been evolutionarily optimized to grow and function inside a 3D gel‐like microenvironment with specific biochemical and biophysical cues, governed by the biochemistry and mechanical properties of the surrounding extracellular matrix (ECM). A scaffold can help recapitulate the natural microenvironment of the cells, which is crucial for cell behavior^[^ ^24^ ^]^ because the ECM influences cell organization into tissues, cell–cell interactions, and cell–matrix interactions. On a 2D surface, most of the cell membrane either interacts with the stiff surface or is exposed to a liquid solution, generating a basal‐apical directionality. This geometry leaves only the perimeter of the cell membrane available for cell–cell interactions and allows the integrins that adhere to the surface to attach only at the bottom of the cell. In 3D culture, cell–cell and cell–matrix interactions can occur on the entire surface of the cell membrane. Exposure to high shear stress from the flowing cell culture media can have a negative effect on cell viability.^[^ ^25^ ^]^ Scaffolding of 3D cultures can reduce or regulate shear stress by a protective soft and elastic surrounding gel or by the porous scaffold wall architecture.^[^ ^26^, ^27^ ^]^ Lastly, cells communicate using gradients, but these gradients are usually lost in 2D cultures due to media mixing, which affects cell motility and 3D organization. Growing cells in an appropriate 3D matrix will have important effects on their biology and behavior, which are likely to translate into a more in vivo‐like tissue structure and improved organoleptic properties. Scaffolds may also have substantial impacts on the scalability and cost‐competitiveness of CM by allowing for the transition of anchorage‐dependent cells to microcarrier‐based suspension cultures.^[^ ^28^ ^]^

If whole‐cut CM is to be created in such a manner as to recapitulate the variety and arrangement of cell types (albeit likely in a simplified form) in genuine animal muscle tissue, the challenges of 3D tissue culture must be overcome in a scalable, cost‐effective, and food‐safe manner. It is also possible that postharvest processing could be used to create products with the desired taste and texture without 3D tissue culture. This review will focus on the challenges related to scaffolding for CM, drawing on literature from CM and adjacent disciplines such as biomedical tissue engineering.

## 2 Hierarchical Structure of Muscle Tissue

To develop practical approaches for muscle tissue engineering, it is necessary to consider the structure of naturally occurring muscle tissue. For CM, understanding how muscle structure relates to meat's nutritional and organoleptic properties is particularly important.

Vertebrates have three classes of muscle (skeletal, smooth, and cardiac). Engineering meat products such as beef, chicken, pork, and fish is essentially skeletal muscle tissue engineering for each species. The structure and function of skeletal muscle tissue (hereafter muscle) are relatively well conserved across species. Therefore the following discussion applies to mammalian, avian, and fish muscle, except where otherwise indicated. This review limits its scope to muscle tissues from vertebrate species, though some of the materials and strategies discussed may also apply to other types of CM, such as shrimp, squid, and foie gras.

**Figure 1:**
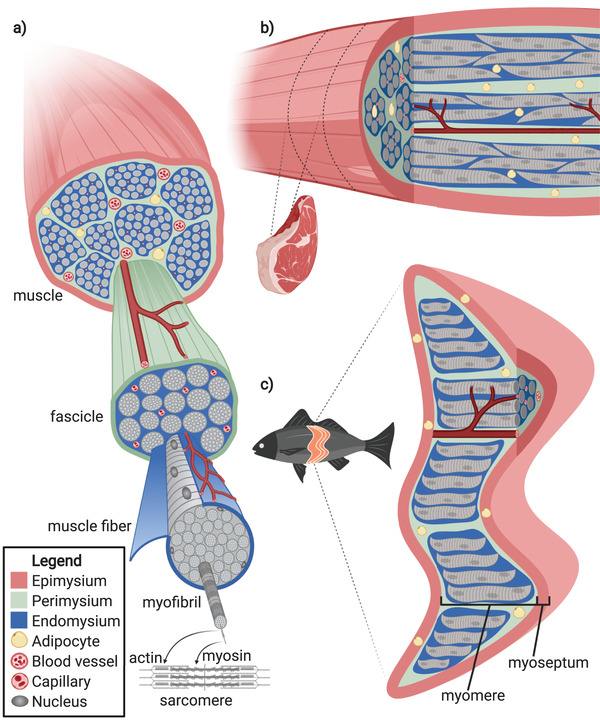
Schematic representation of the hierarchical structure of vertebrate muscle. a) Cross‐sectional view of a muscle with a single fascicle, muscle fiber, myofibril, and sarcomere magnified to show detail. b) Mammalian/avian and c) fish muscle cut longitudinally and in cross‐section to highlight their structural homology. In both panels, a single muscle is oriented such that the muscle fibers run parallel to the page. Dashed lines indicate the relationship between the diagrammed muscle and a steak (b) or a fish (c). Legend applies to all panels.

The fibrous texture associated with meat comes from a complex hierarchical tissue structure. The main functional unit is the muscle fiber (also called a myofiber or muscle cell), which is surrounded by connective tissue, intramuscular fat, vasculature, and nerves. The main determinants of muscle texture and quality are the muscle fibers, fat, and connective tissues.^[^ ^29^ ^]^ Muscle fibers are organized into bundles called fascicles. Connective tissue is separated into endomysium, perimysium, and epimysium, which surround muscle fibers, fascicles, and entire muscles, respectively (**Figure** **1**a). Fish fillets consist of repeating chevron‐ or W‐shaped muscle tissue units called myomeres, each surrounded by connective tissue sheaths called myosepta. Myomeres can be considered equivalent to individual muscles, and myosepta can be considered equivalent to epimysium in terrestrial animals (Figure 1b,c).^[^ ^29^ ^]^

Although the vasculature does not contribute substantially to organoleptic properties, its role in facilitating the transport of oxygen, nutrients, and waste will need to be recapitulated, albeit possibly using a system that is structured quite differently.^[^ ^30^, ^31^, ^32^ ^]^ Reproducing the nerves found in muscle is similarly unlikely to be necessary from an organoleptic standpoint but may contribute to muscle fiber maturation.^[^ ^33^, ^34^ ^]^

The nutritional value of meat arises mainly from its role as a source of high‐quality protein containing all of the essential amino acids, essential fatty acids, and a variety of vitamins and minerals. Therefore, CM manufacturers should aim to recreate a tissue primarily composed (≈90% by volume) of mature muscle fibers surrounded by a small amount (≈10%) of fat and connective tissue^[^ ^29^ ^]^ to recreate the nutritional and structural aspects of meat.

The following sections will discuss the components of muscle tissue likely to be most important to replicate in cultivated meat—muscle fibers, fat, and ECM—as well as common techniques for measuring mechanical properties relevant to meat quality.

### 2.1 Muscle Fibers

A mature terrestrial muscle fiber is very long compared to most cells in the body, at 1–40 mm in length^[^ ^35^ ^]^ and 10–50 µm^[^ ^35^ ^]^ or 10–100 µm^[^ ^29^ ^]^ in diameter. Muscle fibers in fish are considerably shorter, typically only a few mm, with each fiber spanning the distance from one myoseptum to the next.^[^ ^29^ ^]^ The muscle fibers in both groups are mostly multinucleated, with as many as 100 nuclei.^[^ ^35^ ^]^ These cells are packed with cable‐like myofibrils of 1–3 µm diameter, which are bundles of contractile filaments composed of long chains of actin and myosin. These filaments are divided into functional contractile units called sarcomeres. The pattern of overlapping actin and myosin within the myofibrils gives muscle cells their characteristic striated appearance.^[^ ^29^, ^35^ ^]^

Red muscle tissue (as compared to white) is higher in myoglobin and therefore heme iron, making red muscle more nutritious as a source of bioavailable iron.^[^ ^29^ ^]^ However, it has been suggested that heme iron consumption may increase cancer risk,^[^ ^7^ ^]^ making it difficult to definitively label either red or white meat as the “healthier” option. Myoglobin is found within the muscle fibers, primarily in oxidative (slow‐twitch) fibers rather than glycolytic (fast‐twitch) fibers.^[^ ^29^ ^]^ Whereas in the muscles of terrestrial animals, the oxidative and glycolytic fibers tend to be somewhat evenly mixed, glycolytic fibers in fish show a strong spatial separation, typically appearing as a stripe along the animal's side.^[^ ^29^ ^]^

### 2.2 Intra‐ and Intermuscular Fat

Intramuscular fats are a key determinant of meat juiciness, flavor, and nutrition^[^ ^29^ ^]^ and contribute to tenderness through various mechanisms.^[^ ^36^ ^]^ Intramuscular fat is composed primarily of adipocytes, which are found embedded in the muscle tissue between both muscle fibers and fascicles. Intramuscular fat also includes structural lipids, phospholipids, and intracellular lipid droplets within muscle fibers.^[^ ^29^ ^]^ Fat is nutritionally important as a source of the lipophilic vitamins A, D, K, and E and essential lipids such as omega‐3 polyunsaturated fatty acids.^[^ ^37^ ^]^ Overall, saturated fat content is typically higher in beef and pork *Longissimus* muscles than in fish and avian meats, which have higher percentages of polyunsaturated fatty acids.^[^ ^37^ ^]^ The content of protein, total fat, and saturated, monounsaturated, and polyunsaturated fatty acids are shown for some example meat products in **Table** **1**.

**Table 1:** Protein, fat, and fatty acid content of some example meat products.^[^ ^38^ ^]^

Product category	FoodData Central product description (ID)	Protein [g/100 g]	Total lipid (fat) [g/100 g]	Protein:fat ratio	Fatty acids, total saturated [g/100 g] (% of total fatty acids)	Fatty acids, total monounsaturated [g/100 g] (% of total fatty acids)	Fatty acids, total polyunsaturated [g/100 g] (% of total fatty acids)
Beef	Beef, grass‐fed, strip steaks, lean only, raw (169429)	23.1	2.69	9:1	1.03 (48%)	0.995 (47%)	0.108 (5%)
Pork	Pork, fresh, loin, top loin (roasts), boneless, separable lean only, raw (168315)	22.4	4.06	6:1	1.25 (38%)	1.6 (49%)	0.409 (13%)
Poultry	Chicken, broiler or fryers, breast, skinless, boneless, meat only, raw (171077)	22.5	2.62	9:1	0.563 (34%)	0.689 (41%)	0.424 (25%)
Fish (lean)	Fish, tilapia, raw (175176)	20.1	1.7	12:1	0.585 (40%)	0.498 (34%)	0.363 (25%)
Fish (fatty)	Fish, salmon, sockeye, raw (173691)	22.2	4.69	5:1	0.814 (25%)	1.37 (41%)	1.12 (34%)

Intramuscular fat content varies according to factors including species, type of muscle, breed, and nutritional intake.^[^ ^37^ ^]^ The average size of animal adipocytes is also heterogeneous and dependent on intracellular lipid accumulation. For instance, salmon adipocytes from the myosepta of white muscle have variable sizes though most have diameters of less than 50 µm,^[^ ^39^ ^]^ while bovine intramuscular adipocytes of “high‐marbling” breeds such as Japanese Black have average adipocyte diameters of >100 µm at 26 months of age.^[^ ^40^ ^]^

In fish such as rainbow trout and red seabream, lipid depots can be found in the myosepta of white muscle, between muscle fascicles, in red muscle, and subcutaneously, while Pacific herring and Pacific saury also accumulate lipids in these regions but possess more pronounced fat accumulation in red muscle and subcutaneous sites.^[^ ^41^ ^]^ In Atlantic salmon, high numbers of adipocytes are located in the myosepta of red and white muscle.^[^ ^42^ ^]^ Additionally, intracellular lipid deposition has been reported within both types of muscle cells, though more apparently in red muscle.^[^ ^42^ ^]^

### 2.3 The Extracellular Matrix

**Figure 2:**
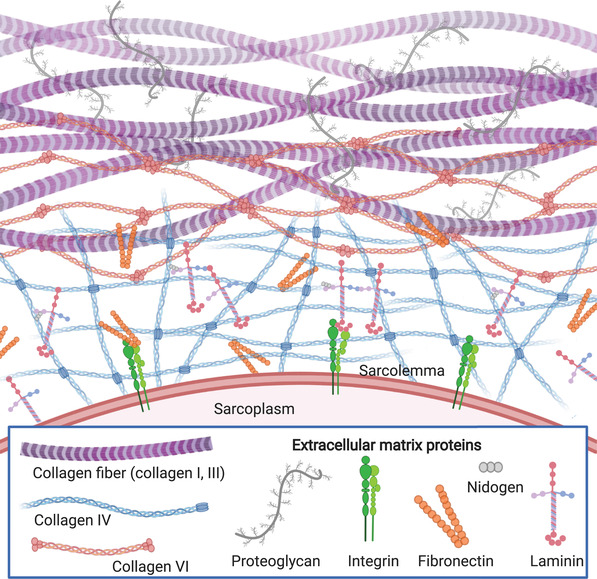
Schematic representation of the composition of the extracellular matrix.

Muscle fibers are embedded in a dense connective tissue composed of many ECM molecules, several of which interface directly with the muscle fibers (**Figure** **2**).^[^ ^43^ ^]^ It has been suggested that distinctions between endomysium, perimysium, and epimysium are relatively arbitrary based on microscopic observations, which reveal that distinct structural stratification may not be accurate.^[^ ^43^ ^]^ Even so, some consistent differences in the molecular makeup of the three connective tissue layers have been observed. The endomysium, which surrounds individual muscle fibers, is distinguished by a mechanically strong collagenous network. The mechanical strength arises from the network structure rather than the properties of individual collagen fibers. The majority of muscle's load‐bearing ability arises from this dense ECM and not the muscle fibers themselves, revealing the importance of a strong support structure for mature muscle cells.^[^ ^43^ ^]^ Therefore, recapitulating the mechanical properties of the ECM, whether by using mechanically similar scaffolding materials or by inducing cells to secrete their own ECM, will be necessary for CM to achieve the texture of conventional meat. Bodiou et al. estimate that muscle‐like stiffness is in the range of 2–12 kPa, which could be beneficial for muscle progenitor cell expansion, and that increasing the tissue stiffness could induce muscle differentiation.^[^ ^44^ ^]^ However, tissue is nonhomogenous, and stiffness variations of the different ECM components are important for cell behavior and final texture. Hydrogels with temporal tunable stiffness could show merit in that respect.^[^ ^45^ ^]^ Indeed, the ECM has profound effects on conventional meat's quality, both through its biological effects on muscle fibers in vivo as well as through changes during postmortem aging.^[^ ^46^ ^]^ Some key molecular components of the ECM—collagen, proteoglycans, and glycoproteins—will be discussed in detail in the following sections.

#### 2.3.1 Collagen

Collagen is the main structural protein in muscle ECM. While fibrillar collagens I and III predominate in mammalian muscle, collagens I and V are the main collagens in fish muscle.^[^ ^29^, ^47^ ^]^ Other collagens present include types IV, V, VI, XI, XII, XIV, XV, and XVIII.^[^ ^43^ ^]^ The muscle basement membrane is primarily composed of a network of collagen IV, but types VI, XV, and XVIII are also present, where the latter two are heparan sulfate proteoglycans.^[^ ^43^ ^]^ Collagen cross‐linking contributes substantially to the mechanical properties of muscle tissue and can vary greatly depending on muscle type, species, and age.^[^ ^29^ ^]^ Collagen‐rich muscles are slightly less nutritious due to the increased quantity of glycine, a nonessential amino acid,^[^ ^29^ ^]^ and the lack of the essential amino acid tryptophan.^[^ ^48^ ^]^

In chicken and pork, it is generally assumed that the effects of differences in collagen levels (within the typically occurring range) on sensory quality are limited.^[^ ^29^ ^]^ This is because most animals raised for conventional meat are consumed when they are fairly young when collagen cross‐linking levels are still quite low. In fish, muscle collagen has low thermal stability and therefore cannot maintain its structure during cooking. Therefore, the texture of fish meat is mainly attributed to the muscle fibers.^[^ ^29^ ^]^ An important consequence of this lower thermal stability is that fish muscle becomes flaky when cooked due to the melting of collagen. Scaffolds for cultivated fish will need to recapitulate this lower thermal stability either by having a lower melting temperature themselves or by providing an environment conducive to the secretion of appropriate collagens, together with degradation of the original scaffold, if the cooked product is to have the appropriate texture.

#### 2.3.2 Proteoglycans and Glycoproteins

Proteoglycans and glycoproteins are important structural components of the ECM. Both macromolecules contain a protein bound to carbohydrate but differ based on the type of carbohydrate. Proteoglycans are composed of a protein core with attached chains of negatively charged and highly hydrated glycosaminoglycans such as heparan sulfate and dermatan sulfate.^[^ ^43^ ^]^ Proteoglycans help to link the basement membrane to the collagenous endomysium by binding directly to collagen.^[^ ^43^ ^]^ They also function to sequester growth factors. The most abundant proteoglycans in muscle tissue are those that bind to the glycosaminoglycans dermatan sulfate and chondroitin sulfate, including decorin and biglycan.^[^ ^43^ ^]^ Oligosaccharide‐containing glycoproteins such as fibronectin, laminin, and nidogen help to link the muscle fiber cell membrane to the basement membrane. Fibronectin and laminin bind directly with integrins at the cell surface and to collagen IV in the basement membrane. The glycoproteins also bind to each other, contributing to the complex interlocking network structure of the basement membrane.^[^ ^43^ ^]^

#### 2.3.3 Dynamic Nature of the Extracellular Matrix

The ECM is constantly being synthesized, degraded, and reorganized. ECM degradation is necessary for myotube formation and cell migration, and the turnover is largely regulated by catabolic enzymes called matrix metalloproteinases (MMPs) and their inhibitors (tissue inhibitors of metalloproteinases, or TIMPs). The gelatinases (MMP‐2 and ‐9) degrade collagen IV, fibronectin, laminin, and the proteoglycans, and the collagenases (MMP‐1 and ‐13) degrade collagens I and III.^[^ ^43^ ^]^ Within muscle, ECM is primarily secreted and maintained by fibroblasts. Myogenic cells have been shown to secrete collagen, decorin, and MMP‐2, and satellite cells secrete MMP‐2, but fibroblasts are necessary to organize the ECM molecules into the proper matrix structure.^[^ ^43^ ^]^ Given the critical role of MMPs and TIMPs in cellular differentiation, migration, and proliferation,^[^ ^49^ ^]^ these enzymes may serve as attractive cell line engineering targets to optimize downstream CM manufacturing processes.

### 2.4 Quantifying Texture

Together, the aligned muscle fibers, fat, structured connective tissue, and other structural elements of a muscle give rise to its mechanical properties, which are perceived as texture if the muscle is eaten as meat. A variety of instrumental methods are commonly used to measure texture, and it can be difficult to make robust comparisons between data generated by different laboratories.

Two of the most common methods for assessing meat tenderness and other textural properties include the Warner–Bratzler shear force (WBSF) and texture profile analysis (TPA) methods.^[^ ^50^ ^]^ In the WBSF method, the force required to cut through a sample using a V‐shaped blade is measured. TPA measures the force and deformation during two successive compressions of a sample, simulating two biting actions to calculate hardness, springiness, adhesiveness, and cohesiveness, as well as additional parameters derived from these.^[^ ^50^ ^]^ Previous studies have attempted both to define WBSF thresholds corresponding to the perception of meat as “acceptably” tender and to determine how closely this measurement correlates with the perception of tenderness. As reviewed by Holman and Hopkins, WBSF tends to be negatively but imperfectly correlated with tenderness and to explain a proportion (22.5% in beef and 48% in lamb) of variation in tenderness across samples. Proposed thresholds for beef vary substantially, though they often tend to be in the range of ≈40 N.^[^ ^51^ ^]^ Both WBSF and TPA suffer from limitations because the measured values are heavily dependent on experimental conditions, and these are not always consistent across laboratories,^[^ ^50^ ^]^ which may explain some of the variability in the WBSF thresholds identified across studies. It has been suggested that TPA is more reliable for measurements on raw samples, whereas WBSF may be preferable for cooked samples.^[^ ^50^ ^]^ However, when comparing both methods in cooked and uncooked meat in relation to sensory analysis, TPA of cooked meat was found to be the best predictor of meat texture parameters.^[^ ^52^ ^]^ TPA better predicted juiciness and hardness, which is considered to be most valued by consumers, and WBSF was found to be useful for predicting springiness. Fish et al. designed a custom device for measuring the WBSF of small samples needed for CM R&D.^[^ ^53^ ^]^

Another commonly reported metric is Young's modulus or modulus of elasticity, which indicates the amount of stress (i.e., force per unit area) required to stretch or compress a substance.^[^ ^54^ ^]^ It is important to note that for anisotropic samples like meat, Young's modulus, and other mechanical properties may be substantially different depending on the orientation in which the sample is measured. For example, Takaza et al. reported stress values over sevenfold higher for pig muscle samples stretched in the transverse direction than those stretched by the same amount longitudinally.^[^ ^55^ ^]^ Meat samples may also show heterogeneous properties within a single sample even when measured in the same direction relative to the direction of the muscle fibers. To account for this heterogeneity, Boots et al. measured Young's moduli in a grid across the surfaces of meat samples.^[^ ^56^ ^]^

More recently, methods have been investigated for measuring the physical properties of meat through nondestructive methods such as MRI and ultrasound.^[^ ^57^, ^58^ ^]^ **Table** **2** shows some examples of instrumental texture measurements taken from meat samples from various species.

**Table 2:** Published instrumental texture measurements from a variety of meat products. Products are raw unless otherwise specified. Measurements have been converted to common units where possible. Because comparisons between studies are complicated by differences in experimental setup, the numbers presented here are intended only to give general ranges for typical values

Product category	Specific product	Young's modulus [kPa]	Shear force [N]	TPA hardness/firmness [N]	TPA resilience [%]
	Beef Tenderloin^[^ ^59^ ^]^	227.6 ± 82.9		2.4 ± 0.8	8.2 ± 1.3
	72 h postmortem *Longissimus* muscle from Japanese black steers aged 8–32 mo, measured in transverse direction^[^ ^60^ ^]^		3.4–4.2 (measured with straight‐edged blade of 0.35 mm thickness)		
Beef	72 h postmortem *Semitendinosus* muscle from Japanese black steers aged 8–32 mo, measured in transverse direction^[^ ^60^ ^]^		4.4‐5.9 (measured with straight‐edged blade of 0.35 mm thickness)		
Pork	Bacon^[^ ^59^ ^]^	59.2 ± 23.6		0.7 ± 0.2	34.6 ± 3.7
	Pork loin frozen at 24 h postmortem at −20 °C, then cooked to a core temperature of 75 °C prior to testing, measured in transverse direction^[^ ^61^ ^]^		51.58 ± 16.2 (WBSF)	29.54 ± 5.37	
	Fresh (prerigor) *Longissimus dorsi* muscle, measured in transverse direction^[^ ^55^ ^]^	70 (77 kPa stress at stretch 1.1)			
	Fresh (prerigor) *Longissimus dorsi* muscle, measured in longitudinal direction^[^ ^55^ ^]^	9.1 (10 kPa at stretch 1.1)			
	Turkey^[^ ^59^ ^]^	169.4 ± 95.1		5.3 ± 0.2	23.6 ± 0.4
	Chicken breast deboned 2 h postmortem, then cooked to a core temperature of 78 °C prior to testing, measured in transverse direction^[^ ^62^ ^]^		107 ± 46 (WBSF)		
Poultry	Chicken breast deboned 24 h postmortem, then cooked to a core temperature of 78 °C prior to testing, measured in transverse direction^[^ ^62^ ^]^		37 ± 12 (WBSF)		
Fish (unspecified)	Processed Fish Ball^[^ ^59^ ^]^	150.4 ± 53.0		1.5 ± 0.2	48.3 ± 3.1
Fish (fatty)	4 d postmortem Atlantic salmon, measured in transverse direction^[^ ^63^ ^]^			8–18	

## 3 Cultivated Meat

Although substantial progress has been achieved in recent years by both for‐profit companies and academic groups, CM remains a relatively new area of investigation. As might be expected for a nascent field that draws heavily on knowledge gained from previous work aimed at a distinct set of problems, review, and perspective papers about CM (at least 50 by our estimate)^[^ ^10^, ^11^, ^12^, ^13^, ^14^, ^16^, ^18^, ^23^, ^28^, ^37^, ^44^, ^64^, ^65^, ^66^, ^67^, ^68^, ^69^, ^70^, ^71^, ^72^, ^73^, ^74^, ^75^, ^76^, ^77^, ^78^, ^79^, ^80^, ^81^, ^82^, ^83^, ^84^, ^85^, ^86^, ^87^, ^88^, ^89^, ^90^, ^91^, ^92^, ^93^, ^94^, ^95^, ^96^, ^97^, ^98^, ^99^, ^100^, ^101^, ^102^, ^103^, ^104^, ^105^, ^106^, ^107^, ^108^, ^109^ ^]^ currently outnumber original research papers where CM production is the primary intended application (at least 24)^[^ ^15^, ^59^, ^110^, ^111^, ^112^, ^113^, ^114^, ^115^, ^116^, ^117^, ^118^, ^119^, ^120^, ^121^, ^122^, ^123^, ^124^, ^125^, ^126^, ^127^, ^128^, ^129^, ^130^, ^131^ ^]^ or CM‐related patents (at least 24).^[^ ^132^, ^133^, ^134^, ^135^, ^136^, ^137^, ^138^, ^139^, ^140^, ^141^, ^142^, ^143^, ^144^, ^145^, ^146^, ^147^, ^148^, ^149^, ^150^, ^151^, ^152^, ^153^, ^154^, ^155^ ^]^ These estimates do not include LCA or TEA studies, consumer acceptance studies, or purely computational analyses. The following sections will summarize the necessary considerations related to choosing a tissue engineering strategy for use in cultivated meat and outline the general process by which cultivated meat is likely to be produced.

### 3.1 Criteria for Cultivated Meat Production

The abundance of existing tissue engineering research is a major advantage for the emerging field of CM. However, the fields of biomedical tissue engineering and CM apply similar solutions to very different problems. The two fields operate with very different constraints in place, so innovators intending to bring CM to market will need to solve additional challenges to turn engineered tissues into viable food products. Some of these differences are qualitative (e.g., CM needs to taste good but does not need to be capable of integrating with the vasculature of a host) and others are merely quantitative (e.g., cost is a consideration for both fields, but the requirements for CM are much more stringent). Some of these differences are summarized in **Table** **3**.

**Table 3:** Key differences between cultivated meat and biomedical tissue engineering likely to impact the choice of scaffolding biomaterials

Attribute	Cultivated meat	Biomedical tissue engineering
Primary purpose of tissue construct	Texture, flavor, color, cooking properties, and nutritional value of the final product are key attributes and must accurately mimic those of conventional meat. These characteristics may be influenced by the presence, organization, and function of contractile proteins,^[^ ^29^ ^]^ but these attributes are otherwise unimportant. Intramuscular fat is necessary for its effects on flavor and texture.^[^ ^37^ ^]^	The construct must be biocompatible, capable of integration into the host tissue, and once implanted must exhibit the appropriate biological functionality. In the case of muscle tissue, the ability to contract in response to neural input is a necessary feature.^[^ ^156^ ^]^
Cost and scale	Products will be produced on a large scale, and high costs would severely limit the potential market size. While moderately expensive products may be successful for early market entry or niche product categories, the ultimate goal is price parity with commodity meat. Reliable, low‐cost, and scalable supply chains for scaffolding materials and other inputs will be required.^[^ ^93^ ^]^	A high degree of customization is required, often including autologous cells. Thus, implanted tissue‐engineered constructs are typically one‐of‐a‐kind. Expensive scaffolding and other materials and high production costs are more tolerable.
Postharvest survival	Postharvest viability is not important except insofar as it influences the food‐relevant properties of the product.	Engineered tissues must maintain long‐term viability and functionality after harvest and implantation.
Suitability of synthetic or animal‐derived materials	The use of synthetic materials is limited due to the need for edible or rapidly biodegradable scaffolds unless the material is completely removed from the final product.^[^ ^104^ ^]^ Animal‐derived materials are generally considered unacceptable, though recombinant versions of the same materials may be an attractive option. Ingredients must be food grade.	Synthetic materials are attractive due to their ability to precisely control their properties and stimulus‐responsiveness. Animal‐derived materials are considered acceptable and are often desirable due to their biocompatibility.^[^ ^156^ ^]^
Degradation profile of scaffold materials	Scaffolds must either be rapidly biodegradable during the differentiation and maturation phase or be edible. The scaffold and its breakdown products, if present in the final product, must be nontoxic even when consumed on a regular basis and must not negatively impact flavor or texture.^[^ ^104^ ^]^ If the scaffold is intended to remain in the final product, its textural properties should mimic the ECM, including changes exhibited during the cooking process.	Biodegradable materials are desirable, but a slow degradation process is acceptable.^[^ ^157^, ^158^ ^]^ The scaffold or its breakdown products must not be harmful to the patient. For materials that would be problematic in larger quantities, the dosage can be accurately predicted.
Target species	The primary target species are common livestock species (e.g., cow, chicken, pig) and commonly consumed seafood species (e.g., shrimp, salmon, tuna). Target species may also include others that are less commonly eaten today but desirable from a culinary perspective, so there is a need to develop methods that can be applied across a wide variety of species.	Humans are the primary target species, with some research also occurring in rodents and other mammalian model species. Biomedical tissue engineering research focused on birds, fish, crustaceans, and other groups is essentially nonexistent.
Immune‐related considerations	Common allergens should ideally be avoided in media and scaffolds, and they must be clearly labeled if used. Because the product is intended to be eaten rather than implanted, immune‐related considerations are largely similar to those for other food products.	The construct must be able to be implanted without triggering a harmful immune response. This challenge necessitates consideration of potential immune or fibrotic responses to the scaffold and makes the use of patient‐derived cells an attractive option.^[^ ^156^ ^]^
Harvesting methods	Intact, differentiated CM products must be harvested at high frequency and in large volumes. Existing technologies for harvesting large volumes of cells such as centrifuges may not be appropriate for differentiated tissues or whole‐cut products. Novel harvesting technologies that integrate into CM bioreactors and bioprocesses, are amenable to scale and automation, and that consider food safety and packaging are needed.^[^ ^146^ ^]^	Engineered tissues for transplant are likely to be harvested in low volumes and may require customization depending on patient population or application. There is a low tolerance for tissue or scaffold damage during harvesting. Thus, stimuli‐responsive interfaces may be favorable to enzymatic or mechanical harvesting methods.^[^ ^159^ ^]^ However, these methods may constrain harvesting to thin tissues.
Oxygen, nutrient, and waste transport	During the culture period, the tissue construct must be sufficiently permeable to oxygen, nutrients, and waste products in order to support cellular metabolism. A cell‐based vasculature is not a requirement.^[^ ^123^ ^]^ The tissue's metabolic demands and environmental conditions are more predictable and controlled than what in vivo muscle tissue would encounter.	Vascularization of the tissue construct is necessary to maintain viability before and after implantation and connection to the host vasculature.^[^ ^160^, ^161^ ^]^ The tissue must be sufficiently vascularized to support the levels of metabolic activity expected in vivo, even during bursts of activity.

A key consideration for manufacturing CM will be the fabrication approach. In top‐down approaches, a prefabricated scaffold is populated with cells, which is then perfused to allow cell migration and ECM formation. In bottom‐up approaches, smaller modular units such as cell‐laden sheets, tubes, spheres, organoids, or other microstructures can be used as building blocks for assembly.^[^ ^162^ ^]^ Combining these approaches may also be used to fabricate complex meat products.

The selection of fabrication approach will be dependent on a variety of parameters such as the type of product, how structured that product is, the product's texture, and how amenable the approach is to commercial scale‐up. While many fabrication methods have been conceived of at the laboratory scale, it is unclear which of these may be best translated into industry. Some approaches that are effective for biomedical applications will likely be impractical for CM, and others that have proven insufficient in the biomedical realm may be worth reconsidering for food production. Current approaches are reviewed in further detail throughout.

### 3.2 The General Production Process

The production process for CM can be broadly divided into four main areas.^[^ ^68^ ^]^ First, cell lines capable of differentiating into muscle fibers, adipocytes, and a handful of other important cell types that make up meat (such as fibroblasts) need to be developed from the species of interest.^[^ ^97^, ^163^ ^]^ Second, media formulations that support high rates of proliferation using low‐cost, food‐safe ingredients need to be developed.^[^ ^92^, ^113^, ^114^, ^164^, ^165^ ^]^ Third, at least for certain bioprocesses and product types, food‐safe scaffolds that mimic the function of the ECM need to be developed.^[^ ^44^, ^104^, ^118^, ^119^, ^123^ ^]^ Finally, bioreactors and bioprocesses must be developed and scaled, keeping in mind constraints related to cost, sterility, food safety, and the ability to maintain appropriate conditions for long‐term cell growth and tissue maturation.^[^ ^83^, ^93^, ^128^ ^]^ While the biopharmaceutical industry cultures mammalian cells in stirred‐tank bioreactors up to 20 000 L,^[^ ^166^ ^]^ substantial innovation in bioreactor design is still needed, particularly concerning the ability to support large‐scale production of structured tissues. Depending on the desired end product and the chosen production process, there may be additional postharvest processing steps required to form the cells or tissues into the final product. These processes may vary dramatically between products, and in many cases may be similar to the methods used to process conventional meat.

**Figure 3:**
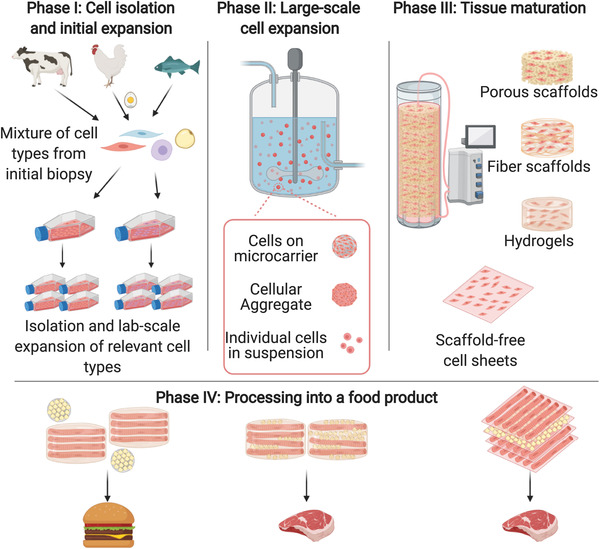
Schematic of the general production process for cultivated meat.

In practice, the details of the production process may differ substantially for different companies and products, but **Figure** **3** shows a general outline of what some potential production processes might look like. The initial phase in any production process will be to isolate and characterize appropriate cells from the species of interest and bank these cells for future use. In many cases, this step will encompass the development of a stable, immortalized cell line. Companies may undertake the cell isolation and cell line development steps themselves or license an existing line. In phase II, cells are expanded to increase total biomass. The goal is to produce a large number of cell doublings while keeping the cells in an undifferentiated, and therefore proliferative, state. In this example, cells are grown in a stirred‐tank bioreactor and may be grown on microcarriers, as aggregates, or as single cells. In phase III—tissue maturation—cells are grown under conditions that promote differentiation and maturation of the cells, typically but not always on scaffolds. The choice of media and bioreactor are crucial in both phases II and III and will likely differ between the two phases. For some product types, a final processing step will be necessary to transform the engineered tissues into a final product. For example, scaffolds laden with mature myofibers might be combined with edible microcarriers on which adipocytes have been differentiated in a separate phase III process to form a burger patty. Alternatively, multiple cell types might be differentiated on the same scaffolds, which are then combined to form the final product, or scaffold‐free sheets (in which secreted ECM proteins take the place of the scaffold) may be stacked. Additional variations on this general scheme might combine phases II and III or might avoid the need for phase IV by maturing the tissues on a larger scaffold that is harvested and directly processed in the same manner as conventional meat.

## 4 The Basic Scaffold Types

**Figure 4:**
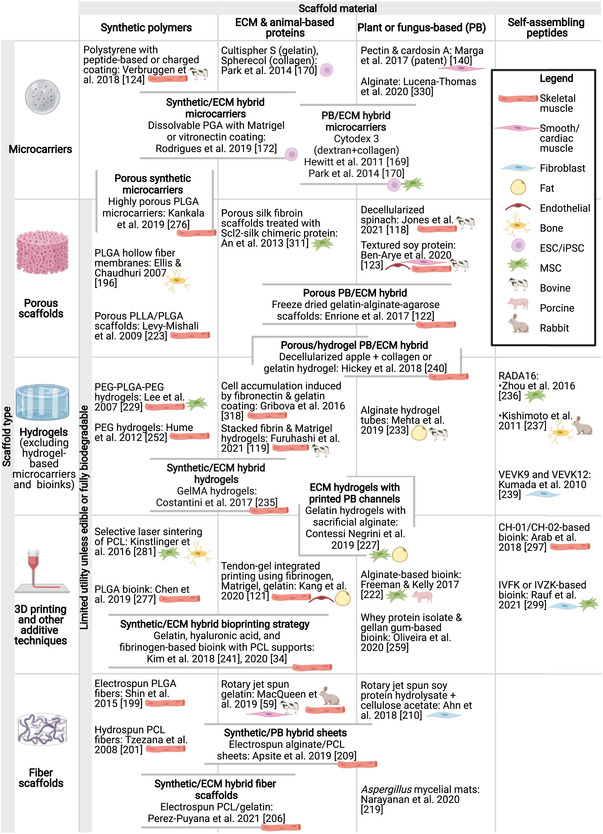
Summary of scaffold types and materials. Gray outlined items indicate scaffolds belonging to more than one category. Scaffolds that have been tested with cell types or cells from species relevant to cultivated meat are indicated with icons to the lower right of the entry. The “plant or fungus‐based” category also includes molecules commonly produced by these groups that are also sourced from other organisms (e.g., cellulose from bacteria or algae, or chitosan from crustaceans). For a more comprehensive version of this figure, please see Figure S1 of the Supporting Information.

Scaffolds for cell culture and tissue engineering come in a variety of forms (**Figure** **4**, Figure S1, Supporting Information, and **Table** **4**). The simplest scaffolds are microcarriers, typically used for large‐scale cell proliferation. For tissue maturation, most scaffolds can be categorized as porous scaffolds, hydrogels, or fiber scaffolds. While both porous and fiber scaffolds contain void spaces through which media can circulate, they differ in structure, with porous scaffolds having a sponge‐like structure and fiber scaffolds being composed of long, thin fibers. Typically, fiber scaffolds are produced by electrospinning, but highly similar structures applicable to tissue engineering are also produced by certain species of fungi. In some cases, microcarriers also belong to one of the other three categories, most commonly hydrogels. However, due to their unique role as a scaffold for suspension culture, they are discussed separately from the other scaffold types. Additive methods such as 3D bioprinting are also common and often use hydrogel‐based bioinks. Like microcarriers, additive methods are associated with a distinct set of considerations compared to other strategies and therefore are discussed separately. Scaffold‐free approaches such as cell sheet‐based methods may also be used.

**Table 4:** General advantages and disadvantages of various strategies for scaffolding or tissue structuring

Scaffold type	Advantages	Disadvantages and uncertainties
Microcarriers	Microcarriers are simple to produce and allow for efficient scale up of adherent cells.	The use of nonedible microcarriers will require a dissociation step, which is likely to increase costs and could introduce safety concerns.
Porous scaffolds	Porous scaffolds can be produced relatively easily and cheaply and provide a robust structure for cell growth, and the distribution of pore sizes can be tuned to simultaneously bias cellular differentiation in the intended direction as well as facilitate sufficient oxygen, nutrient, and waste transport.	While porous scaffolds can display some degree of anisotropy, thereby facilitating muscle fiber alignment, many types of porous scaffold may be less well‐suited than fiber scaffolds to the introduction of strong and large‐scale directionality to the tissue, as would be desired for some whole‐cut meat products.
Fiber scaffolds	Fiber scaffolds are well‐suited to the task of inducing muscle fiber alignment, and it is relatively straightforward to produce a scaffold with strong anisotropy across the entire construct. Spinning techniques can be applied to a variety of biomaterials.	There is a need for the development of highly scalable versions of spinning techniques suitable for production of scaffolds at the cost and scale demanded by the CM industry.
Hydrogels	Hydrogels mimic the structure of the naturally occurring ECM, which is an advantage for cellular compatibility. In many cases, they also require little scaffold material in relation to the size of the construct, meaning that higher costs on a dry weight basis may be tolerable.	Bulk hydrogels, i.e., those cast in solid form and not used as a source material for cryogel‐based porous scaffolds or for bioprinting, do not inherently feature any sort of anisotropy or patterning.
3D bioprinting	Bioprinting can be used to produce defined structures at a variety of scales, with precise control over the positioning of cells and bioinks.	A tradeoff exists between printing resolution and speed, implying that the achievable resolution at scale may be lower than what is possible in theory.
Scaffold‐free approaches	Scaffold‐free approaches based on cell sheets or organoids do not introduce any exogenous scaffold material but rather depend on the cells to secrete their own ECM. This may present advantages from a regulatory perspective and may contribute to better organoleptic and nutritional similarity to conventional meat.	These approaches have thus far been less well studied than the others described here, and therefore there is a higher degree of uncertainty as to their suitability. In particular, scalability may be a significant challenge.

### 4.1 Microcarriers

In 1967, van Wezel first introduced the concept of growing adherent cells on small particles in suspension, demonstrating that adherent mammalian cells could be cultured on commercially available positively charged Sephadex beads.^[^ ^167^ ^]^ Since then, a variety of commercial products have become available, primarily designed for scaling up cells for the pharmaceutical and biomedical industries. These cell types include human mesenchymal stem cells (MSCs),^[^ ^168^, ^169^ ^]^ embryonic stem cells (ESCs),^[^ ^170^, ^171^ ^]^ and induced pluripotent stem cells (iPSCs).^[^ ^172^ ^]^ The microcarriers are composed mainly of polystyrene, cross‐linked dextran, cellulose, gelatin, or polygalacturonic acid (PGA) and coated with collagen, peptides containing adhesion motifs, or positive charges to promote cell adhesion. Their diameters are typically between 100 and 200 µm.^[^ ^44^ ^]^ A recent review^[^ ^44^ ^]^ comprehensively describes existing microcarrier technologies and details how they might be adapted for the CM industry. The authors describe three possible scenarios for how microcarriers could be employed. 1) As a temporary carrier to support cell proliferation, after which the cells are removed and further processed. 2) A temporary carrier that is dissolved or degraded to release the cells. 3) An edible carrier that is incorporated into the final product. The authors further noted that no commercially available microcarriers had been developed specifically for the CM industry, indicating an area of high need as well as potential. Recently, some companies have begun to fill this gap, with both Matrix Meats^[^ ^173^ ^]^ and Tantti Laboratory^[^ ^174^ ^]^ offering edible microcarriers intended for use in CM. SingCell, whose founder holds patents related to microcarriers,^[^ ^175^, ^176^ ^]^ is also using microcarriers to assist CM clients with bioprocess scale‐up and comanufacturing.

Primary bovine myoblasts have been cultured on several commercially available microcarriers and demonstrated to undergo bead‐to‐bead transfer;^[^ ^124^ ^]^ however, the reliance on enzymatic removal from carriers could be cost‐prohibitive for scale‐up because the food industry has much smaller profit margins compared to the pharmaceutical industry. A patent on edible microcarriers made from pectin and cardosin A^[^ ^140^ ^]^ was assigned to Modern Meadow, a company that has positioned itself as a provider of animal‐free biofabricated materials for a variety of industries. While these developments are promising, there is still a wide scope for advancing microcarrier technology specifically for CM. A recent review of microcarrier surface modifications to improve both the attachment and detachment of cells from microcarriers^[^ ^177^ ^]^ describes several promising technological advances. Derakhti et al. emphasized that microcarriers enabling nonenzymatic detachment methods, such as those with thermoresponsive coatings,^[^ ^178^ ^]^ are particularly interesting, although they still require more optimization to improve on the standard enzyme‐based cell recovery methods. Another consideration is optimizing the shape of carriers to shield cells from shear stresses from fluids or bursting bubbles in aerated bioreactors, which was demonstrated using a microfluidic photocrosslinking system to fabricate the hydrogel microcarriers.^[^ ^179^ ^]^ These types of advances reveal how newly developed biomaterials technologies could be translated to assist in CM scale‐up and manufacturing.

While microcarriers offer a relatively simple solution to the problem of how to expand mammalian cells at scale with minimal space requirements, they may also introduce limitations related to the cost of cell dissociation and separation, the cost of the microcarriers themselves, maximum cell densities that can be achieved, and potential impacts on the nutritional and/or organoleptic properties of the final product. Alternative strategies based on spheroids,^[^ ^180^, ^181^, ^182^, ^183^ ^]^ organoids,^[^ ^184^ ^]^ or adaptation to single‐cell suspension culture^[^ ^185^ ^]^ may obviate the need for microcarriers. At this point, it is unclear which of these strategies may prove most effective at producing high‐quality CM products at a sufficiently low cost.

### 4.2 Porous Scaffolds

**Figure 5:**
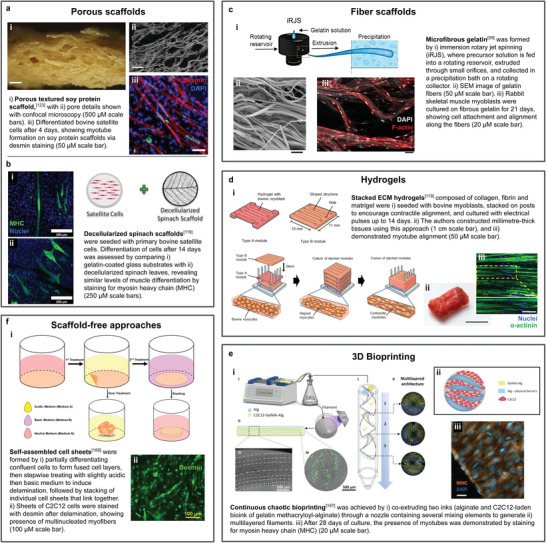
a–f) Highlighted examples of scaffold types and fabrication methods developed by various research groups that are working on cultivated meat. An overview of their approach is demonstrated, as well as images verifying the presence of muscle‐like markers and structures in their chosen cell types grown on or in the constructs. a) Adapted with permission.^[^ ^123^ ^]^ Copyright 2020, Springer Nature. b) Adapted with permission.^[^ ^118^ ^]^ Copyright 2021, Elsevier. c) Reproduced under the terms of the Creative Commons Attribution (CC BY) license.^[^ ^59^ ^]^ Copyright 2019, The Authors. Published by Springer Nature. d) Reproduced under the terms of the Creative Commons Attribution (CC BY) license.^[^ ^119^ ^]^ Copyright 2021, The Authors. Published by Springer Nature. e) Adapted with permission.^[^ ^187^ ^]^ Copyright 2021, Elsevier. f) Adapted with permission.^[^ ^188^ ^]^ Copyright 2020, American Chemical Society.

Scaffolds with a pore size in the range of tens to hundreds of microns^[^ ^186^ ^]^ have a sponge‐like structure that provides the mechanical stability required for seeded cells to form tissues and deposit ECM (**Figure** **5**a,b). For muscle tissue engineering, these scaffolds should recapitulate the structure, mechanical properties, and composition of the perimysium connective tissue,^[^ ^23^ ^]^ taking into account that the scaffold would remain an integral component of the mature tissue.

Ben‐Arye et al. used commercial textured vegetable protein (TVP), a highly scalable and inexpensive porous material generated by extrusion of soy protein powder, which can be generated from a sidestream of the oil industry, as scaffolds for CM^[^ ^123^ ^]^ (Figure 5a). Commercial TVP products were characterized as tissue engineering scaffolds, bovine cell composition and media were optimized, and muscle tissue differentiation and ECM composition were investigated, showing evidence for improved myogenesis by coculture with ECM‐producing supporting cells and complex ECM deposition. Scaffold parameters such as pore size, nutritional value, texture, and elasticity could also be improved by customizing the plant protein composition of the TVP and the extrusion parameters, which are well established for plant‐based meat analogs,^[^ ^189^ ^]^ or by using other porous plant‐based materials such as seitan, other plant protein materials used as ingredients in meat analogs,^[^ ^190^ ^]^ or even bread.^[^ ^191^ ^]^

Commonly used scaffold fabrication techniques such as particle leaching, melt molding, freeze‐drying, and gas foaming^[^ ^166^, ^192^ ^]^ often use synthetic polymers, which should be replaced with edible ones^[^ ^193^ ^]^ for use in CM. For example, Chang et al. produced porous scaffolds by rapidly freezing a solution of gelatin and hyaluronic acid, leading to the formation of ice crystals at the same time as cross‐links were formed throughout the scaffold structure.^[^ ^194^ ^]^ These scaffolds exhibited >90% porosity and were able to support attachment, proliferation, and differentiation of porcine adipose‐derived stem cells,^[^ ^194^ ^]^ though the use of similar scaffolds for CM depends on the development of low‐cost sources of gelatin from recombinant or other animal‐free systems. Newer techniques that improve the resolution of scaffold architecture can optimize tissue quality for regenerative medicine purposes,^[^ ^192^ ^]^ though their applicability to CM may be limited by cost and scale considerations.

Pore size, porosity, and scaffold material are key factors impacting tissue development and cell survival. While pore size is important for cell culture, integration of larger pores suitable for media perfusion should also be considered for pseudo‐vascularization^[^ ^195^, ^196^ ^]^ to allow for efficient transport of nutrients and oxygen in thicker pieces of CM.

### 4.3 Fiber Scaffolds

Spinning techniques, including electrospinning and rotary jet spinning, can be used to produce nanofibers with a variety of useful properties for CM (Figure 5c). These include the ability to support both cell adhesion (to the fibers) and diffusion of oxygen and nutrients (through the spaces between fibers), as well as the ability to produce aligned fibers that may help promote muscle fiber maturation. Porosity values between ≈20% and ≈60% have been reported for rotary jet spun gelatin, indicating the presence of sufficient inter‐fiber spaces to facilitate oxygen, nutrient, and waste transport.^[^ ^59^ ^]^ Spinning techniques can be applied to a variety of materials including polylactic acid (PLA),^[^ ^197^, ^198^ ^]^ poly(lactic‐*co*‐glycolic acid) (PLGA),^[^ ^199^, ^200^ ^]^ poly‐*ε*‐caprolactone (PCL),^[^ ^201^ ^]^ gelatin methacryloyl (GelMA),^[^ ^202^ ^]^ fibronectin,^[^ ^203^ ^]^ albumin,^[^ ^204^ ^]^ and gelatin^[^ ^59^ ^]^ in a relatively high‐throughput manner. Combinations of materials are also common, including PCL and poly(*N*‐isopropyl acrylamide) (PNIPAAm),^[^ ^205^ ^]^ PCL and collagen or gelatin,^[^ ^206^, ^207^, ^208^ ^]^ PCL and alginate,^[^ ^209^ ^]^ and soy protein hydrolysate and cellulose acetate.^[^ ^210^ ^]^

While most investigations of fiber scaffolds for tissue engineering have focused on biomedical applications, MacQueen et al. recently demonstrated the growth of rabbit myoblasts and bovine smooth muscle cells on rotary jet spun gelatin with CM as the intended application (Figure 5c) as well as a histological comparison of the engineered constructs to rabbit muscle, bacon, and ground beef.^[^ ^59^ ^]^ Electrospinning of a combination of PCL and temperature‐sensitive PNIPAAm has been used to produce aligned cell sheets that can be detached from the scaffold by a change in temperature,^[^ ^205^ ^]^ a technique that has been patented^[^ ^211^ ^]^ by a team that includes one of the founders of the cultivated beef jerky and brisket company BioBQ. Furthermore, Matrix Meats, which spun out from a company called Nanofiber Solutions, uses electrospinning technology to create scaffolds. Although most of their patents are based on synthetic polymers and are targeted toward biomedical applications, their portfolio includes an assigned (but not granted) patent on multicomponent electrospun fiber scaffolds^[^ ^212^ ^]^ and a granted patent on aligned fiber scaffolds made out of synthetic or natural polymers including chitosan, collagen, and cellulose.^[^ ^213^ ^]^ Further applications of fibrous scaffolds in CM include those being assessed by Harvard University spin‐out Boston Meats, which aims to innovate on the structure and texture of alternative meat products based on the cofounders’ experience constructing nanofiber scaffolds using ECM proteins and plant‐based materials for muscle engineering and wound healing applications.^[^ ^59^, ^203^, ^207^, ^210^ ^]^

There has been some focus in recent years on developing processes to scale‐up production of electrospun nanofibers, including multijet electrospinning,^[^ ^214^ ^]^ needle‐less approaches implementing a variety of electrode geometries,^[^ ^215^, ^216^, ^217^ ^]^ and high throughput production of core–sheath fibers.^[^ ^218^ ^]^ Certain natural materials exhibit structural properties similar to those produced by spinning techniques. These may offer some of the same desirable characteristics with greater scalability and less need for capital‐intensive processing. For example, mycelial mats from a variety of fungal species produce fibrous structures,^[^ ^152^, ^219^ ^]^ as do certain species of algae.^[^ ^220^ ^]^

### 4.4 Hydrogels

A hydrogel is a hydrophilic polymer matrix with a large water‐holding capacity, where the matrix is cross‐linked by either physical or chemical means (Figure 5d). Most relevant cell types for CM reside inside the ECM, which is itself a type of hydrogel. Therefore, hydrogels are a rational biomaterial choice for tissue engineering of CM. Hydrogels have several vital requirements for tissue engineering. The polymer matrix should be cytocompatible, made of biomaterials that are not toxic for the cells. Because micronutrients and signaling molecules must be able to reach cells throughout the tissue, the hydrogel's diffusion kinetics should allow these molecules to penetrate the entire thickness of the hydrogel at the concentrations and rates demanded for supporting cells. Diffusion kinetics depend on the cross‐linking and microporous structure of the hydrogel.^[^ ^221^ ^]^ The stiffness of the hydrogel can affect cell motility, proliferation, and differentiation.^[^ ^222^, ^223^, ^224^ ^]^ Hydrogels that are too stiff can inhibit proliferation and migration, and cells need to be able to remodel the hydrogel during tissue maturation.^[^ ^225^ ^]^ Optimally, cells will degrade the hydrogel over an appropriate time period and synthesize their own ECM. The biochemistry of the hydrogel is crucial for cytocompatibility. Cell adhesion and proteolytic sites should be incorporated into the hydrogel to facilitate cell adhesion and migration. In addition, the incorporation of growth factor binding molecules such as heparan sulfate is valuable for optimal biochemical signaling.

Hydrogels have several uses for tissue engineering. They can be used as a soft 3D ECM‐like environment,^[^ ^115^, ^119^, ^226^, ^227^, ^228^, ^229^, ^230^, ^231^, ^232^, ^233^, ^234^, ^235^, ^236^, ^237^, ^238^, ^239^ ^]^ as a 3D matrix filler inside porous scaffolds,^[^ ^240^ ^]^ as components of bioinks,^[^ ^34^, ^121^, ^187^, ^222^, ^241^, ^242^, ^243^, ^244^, ^245^, ^246^ ^]^ as thin membranes which may be microstructured to produce alignment of cells,^[^ ^126^, ^247^ ^]^ or as source material to develop porous scaffolds.^[^ ^23^, ^248^, ^249^ ^]^ For the first three uses, cytocompatible gelation is essential, as the cells are introduced into the hydrogel liquid solution before the hydrogel solidifies. Solidification can be achieved using enzymatic gelation,^[^ ^250^ ^]^ thermal gelation within cytocompatible temperature ranges (4–37 °C),^[^ ^251^ ^]^ photopolymerization using cytocompatible durations and wavelengths of exposure, or ionic cross‐linking gelation. Synthetic hydrogels are commonly used for tissue engineering purposes due to their inert biological properties that prevent an immune response. PEG has been used for skeletal muscle tissue engineering^[^ ^252^ ^]^ and can also be found in food products for functional purposes. Food‐grade hydrogels such as carrageenan—derived from several edible species of seaweed—were shown to be suitable for tissue engineering purposes^[^ ^253^ ^]^ and also show merit for CM due to their frequent use in meat processing. Composite hydrogels can better recapitulate the ECM and often show improved properties compared to those composed of a single material. Hyaluronic acid and collagen composite recapitulated the ECM more accurately and was used for 3D adipogenesis.^[^ ^194^, ^225^ ^]^ Similarly, collagen‐fibrin composite provides short‐term (fibrin) and long‐term (collagen) ECM, rendering this composite material more suitable for tissue maturation.^[^ ^254^ ^]^ Hyaluronic acid and alginate composite improved the gelation properties as compared to hyaluronic acid alone.^[^ ^255^ ^]^

### 4.5 3D Bioprinting

Another strategy that is likely to be applicable to CM is 3D bioprinting (Figure 5e), which allows for cells to be deposited in a defined pattern using bioinks with the desired rheological properties.^[^ ^256^, ^257^, ^258^ ^]^ For CM applications, the bioink must be either edible or capable of fully biodegrading during the culture period into edible components. Although 3D printing has been demonstrated for a wide variety of materials, most of the bioinks relevant to CM are those based on hydrogels. Food‐safe, phase‐separated inks composed of a mixture of whey protein isolate and gellan gum have been investigated for their potential use in food bioprinting applications.^[^ ^259^ ^]^ Collagen/gelatin^[^ ^121^, ^187^, ^243^, ^244^ ^]^ and hyaluronic acid,^[^ ^260^ ^]^ both of which are naturally found in mammalian tissues, have also been investigated as bioinks or components of bioinks. Vivax Bio, a subsidiary of 3D Bioprinting Solutions, is focused on CM‐specific applications of the parent company's 3D bioprinting technology.^[^ ^261^ ^]^ The team's scientists have published extensively on tissue engineering and 3D printing,^[^ ^181^, ^183^, ^243^, ^262^ ^]^ including printing of cellular spheroids^[^ ^182^, ^183^ ^]^ and collagen‐based bioinks.^[^ ^243^, ^262^ ^]^

Because of the shear forces exerted on cells when they are printed in a viscous ink, together with the fact that viscosity is necessary for the printed construct to hold its shape, cross‐linkable hydrogels have been investigated as one possible approach to the printing of complex structures while maintaining cellular viability. A method of in situ cross‐linking in which a photo‐crosslinkable methacrylated hyaluronic acid hydrogel is exposed to UV light as it is printed is able to produce structures that hold their shape while maintaining high viability.^[^ ^263^ ^]^ Bioprinting offers certain advantages related to inducing myotube alignment^[^ ^34^, ^241^ ^]^ and the creation of vascular‐like channels,^[^ ^228^, ^242^, ^245^, ^264^ ^]^ which are discussed in more detail in Sections 6.2 and 6.3.

### 4.6 Scaffold‐Free Approaches

Although scaffolds provide many advantages for tissue engineering, including the ability to facilitate oxygen and nutrient transport and precise control over the 3D geometry of the final construct, scaffold‐free methods can also address these challenges (Figure 5f).

Combining multiple planar cell sheets by stacking or other methods can also be used to form 3D tissues. In this case, no scaffold or bioink material at all is used, and cells are held together by their own secreted ECM. The *π*‐SACS method, in which a pH change is used to trigger the delamination of a cell sheet that can then be stacked with additional sheets, was used to form C2C12 constructs several mm in diameter and four cell sheets thick^[^ ^188^ ^]^ (Figure 5f). This method has also recently been explored as a method for cultivating meat from a combination of muscle cells and adipocytes.^[^ ^120^ ^]^ Expected challenges when attempting to use cell sheet‐based methods for CM include the space needed to grow sufficient numbers of cells in 2D culture as well as the labor‐intensive nature of stacking multiple sheets. These challenges could potentially be addressed by novel bioreactor geometries and automated methods for tissue assembly.^[^ ^120^ ^]^

Another scaffold‐free assembly method for CM may be achieved by leveraging cellular self‐organizing principles and advances in organoids. For example, recent studies have shown that tissue‐ or region‐specific organoids can be independently derived and then fused to form functional, complex tissues dubbed “assembloids.”^[^ ^265^ ^]^ In one study, muscle organoids were combined with cortical and spinal organoids to form a functional neuromuscular circuit.^[^ ^266^ ^]^ Similar methods could be used to create larger CM constructs by fusing muscle, adipose, connective, and vascular organoids, for instance. Because organoid fusion can happen on the order of hours and CM does not need to be viable when eaten, assembloids could be constructed and harvested prior to a necrosis event due to nutrient or oxygen deprivation, enabling manufacturers to forego establishing perfused vasculature or pseudovasculature necessary for long‐term cultivation.

## 5 Scaffolding Materials

Selection of appropriate scaffolds for CM will require consideration of a variety of different material properties. **Table** **5** lists some estimates of what appropriate values are likely to be for parameters such as attachment rate, cost, and degradation profile.

**Table 5:** Design criteria for CM scaffolds

Design criterion	Desired value	Rationale
Attachment properties	Attachment rate as high as possible, ideally >90%, for desired cell types; if the material is used as a microcarrier and not included in the final product, efficient and nontoxic detachment methods are required	Large‐scale cell proliferation has been identified as a likely substantial contributor to both the cost and environmental impact of CM production.^[^ ^19^, ^22^ ^]^ Low attachment rates will lead to unnecessary waste of cells, increasing the overall cost and environmental impact, and therefore are not acceptable. Depending on the intended application of the scaffold, it must also be compatible with high proliferation rates and efficient differentiation and maturation. See Section 6.1 for possible strategies aimed at improving attachment.
Cost	As low as possible, ideally <USD $0.10 per kg of final product at scale	To achieve substantial market penetration, CM must be competitive with commodity meat prices of approximately USD $2 per kg.^[^ ^267^ ^]^ Considering the challenges associated with other parts of the bioprocess, it is prudent to assume that scaffolding should contribute very little to the overall cost of production, perhaps <5% of the production cost. The price per kg of scaffold may be higher than this, depending on the mass of scaffold required to produce 1 kg of product. Of course, substantially higher prices may be tolerable in the short term or for premium products.
Degradation profile	Unless edible or removable, complete degradation is required in ≈2–3 weeks, but dependent on the length of the bioprocess and any postharvest aging period	If degradation is intended, it should be completed under the relevant conditions in less time than the culture period plus the postharvest aging period (if any). It is also necessary that the degradation process not leave harmful byproducts in the final product. Any scaffold material that is intended to remain in the product must be suitable as food, i.e., nontoxic and food‐safe with positive or neutral organoleptic and nutritional properties (including after cooking). This requirement does not apply to scaffolds from which the cells will be removed, such as microcarriers.
Porosity	30–90%, or possibly slightly higher if it does not impair mechanical properties of the scaffold; within this range, higher is likely to be better	Zeltinger et al. found that cell seeding for several cell types was more uniform on scaffolds with a porosity of 90% compared to 70%.^[^ ^186^ ^]^ Ben‐Arye et al. demonstrated proliferation and differentiation of myogenic cells on scaffolds with porosities of 42% and 56%.^[^ ^123^ ^]^ MacQueen et al. produced fibrous scaffolds with a porosity of ≈30–55% and were able to successfully grow and differentiate rabbit myogenic cells on said scaffolds.^[^ ^59^ ^]^ Chang et al. demonstrated proliferation and differentiation of adipogenic cells on a 90% porous scaffold.^[^ ^194^ ^]^ In addition, a higher degree of porosity means that a higher scaffold cost per kg may be tolerable, as a smaller mass of a highly porous and therefore less dense scaffold is likely to be necessary for a given mass of final product.
Pore or channel size	50–150 µm pores for myogenic cells, as well as possibly larger 40–400 µm pores for adipogenic cells; pores of 170 µm have been demonstrated to work well Range of channel sizes with a possible range of 4–750 µm, though the lower end of this range may be unnecessary (see Section 6.3.4)	Zeltinger et al. tested for proliferation and ECM secretion of smooth muscle cells and found good performance within a range of 38–150 µm and lower performance at smaller pore sizes, though larger pores were not tested.^[^ ^186^ ^]^ The same study also tested fibroblasts, for which the effect of pore size made little difference, including for scaffolds with pore sizes < 38 µm. Ben‐Arye et al. described the successful proliferation and differentiation of myogenic cells on scaffolds with a wide range of pore sizes, where the majority of individual pores measured <50 µm but over half the total pore area was represented by pores in the range of 50–400 µm.^[^ ^123^ ^]^ Chang et al. demonstrated proliferation and differentiation of adipose‐derived stem cells on a scaffold with a mean pore size of 170 µm. Lower and upper limits for adipose cells were suggested to be on the order of 40 and 400 µm, respectively.^[^ ^194^ ^]^ Miller et al. demonstrated successful media perfusion through hydrogel‐based constructs with branched vascular‐mimicking networks (see Section 6.3) with channel sizes ranging from 150 to 750 µm.^[^ ^228^ ^]^ Vessels in true skeletal muscle are considerably smaller, ranging from 4 µm for capillaries^[^ ^268^ ^]^ to 20–50 µm for arterioles.^[^ ^269^ ^]^ An important feature, especially for larger constructs, will be the presence of channels of a range of sizes.^[^ ^181^ ^]^
Scaffold size and thickness	Product‐dependent, but at least some products will require scaffolds of several centimeters thickness in all directions	Scaffolds intended for processes that include a postharvest fabrication step may be small, but for those where a whole cut of meat is to be grown in one piece, the scaffold will need to be at least the size of the intended product without sacrificing the capacity for oxygen, nutrient, and waste transport.
Young's modulus	≈12–21 kPa for myogenic cells ≈2–3 kPa for adipogenic cells, possibly lower	Boonen et al. observed enhanced maturation of primary myogenic cells cultured on 21 kPa gels as opposed to 3 kPa gels.^[^ ^270^ ^]^ Engler et al. found that the optimal stiffness for myotube differentiation and maturation was 12 kPa, similar to the stiffness of muscle tissue, whereas substrates of 1, 8, or 17 kPa did not support myotube striation.^[^ ^271^ ^]^ Ansari et al. observed better myogenic differentiation of MSCs (as assessed by MyoD expression) cultured on alginate hydrogels of 15 kPa as compared to those of 5, 30, or 45 kPa. Freeman and Kelly found that gels with Young's modulus of ≈5–7 kPa preferentially induced osteogenic rather than adipogenic differentiation in MSCs, whereas softer gels with a modulus of ≈2–2.5 kPa induced approximately equal numbers of cells to adopt each fate.^[^ ^222^ ^]^ Chandler et al. observed better adipogenic differentiation in cells cultured on hydrogels with an aggregate modulus of ≈3 kPa as compared to stiffer gels with a modulus of ≈8 kPa or more.^[^ ^272^ ^]^ Because the test was performed under radial confinement, it can be assumed that the aggregate modulus approximately equals Young's modulus.^[^ ^273^ ^]^
		For anisotropic materials (see Section 6.2), Young's moduli may differ substantially depending on the direction in which the material is compressed, and in these cases much larger Young's moduli may be appropriate.^[^ ^197^, ^200^, ^208^, ^274^ ^]^

Materials commonly used as scaffolding for tissue‐engineered constructs include synthetic polymers, self‐assembling peptides, ECM molecules, and plant‐ or fungus‐derived materials. Multiple materials are often combined to take advantage of each material's useful properties (Figure 4, Figure S1, Supporting Information, and **Table** **6**).

**Table 6:** General advantages and disadvantages of various classes of scaffold biomaterial

Scaffold material	Advantages	Disadvantages and uncertainties
Synthetic polymers	Synthetic polymers have tunable properties and can be used in multiple scaffold configurations. In addition, they have high batch‐to‐batch consistency and low production costs.	Many synthetic materials are not edible or biodegradable, while others have slow degradability or unknown degradation profiles. These materials are currently suitable as microcarriers or other temporary scaffolds, whereas their applicability as integrated scaffolds is uncertain and dependent on the ability to substantially accelerate degradation and ensure food safety.
Self‐assembling peptides	Self‐assembling peptides are able to mimic native ECM architecture and naturally assemble into nanofibrous matrices. They are versatile and can be used for conventional scaffold production methods, as part of bioinks for additive manufacturing, or as coatings to aid in large‐scale cell proliferation.	The application of self‐assembling peptides in CM remains to be assessed in the published scientific literature and is minimally studied for tissue engineering. Current production strategies are costly and could undermine its application in CM.
ECM molecules	ECM molecules offer the most similar environment to naturally occurring tissues and effectively facilitate cell attachment. Combinations of animal‐free ECM proteins and low‐cost plant‐based or other materials may be an effective means of taking advantage of these materials’ desirable properties at a lower cost.	Animal‐derived ECM molecules are unsuited for use in CM, while recombinant expression and other strategies will need to dramatically reduce costs and increase scale to be a viable option for CM production.
Plant and fungus‐derived materials	Some decellularized or minimally‐processed plant materials inherently have structural features that lend themselves to use in CM. For example, the strong anisotropy of celery, green onion bulbs, and grass, the well‐developed vasculature of spinach leaves, and the porous structure of TVP make these materials promising candidates. Alternatively, plant matter can be used in more processed forms, e.g., for electrospinning. Some plants have been shown to effectively support cell adhesion without functionalization.	Decellularization methods will need to be developed that do not substantially increase costs or environmental impacts and that do not introduce safety concerns. The use of plant species is likely to be limited to those that can be produced extremely cheaply and at scale, as many of the plants that have been explored are also ones for which there is a market for direct consumption by humans.

### 5.1 Synthetic Polymers

Synthetic materials do not occur in nature but have distinct features that are advantageous for tissue engineering such as tunability, reproducibility, and being chemically defined.^[^ ^275^ ^]^ However, many synthetic polymers are inedible and have slow degradation rates, meaning that cells would need to be efficiently separated from microcarriers or scaffolds composed of such materials after expansion, and their use as embedded tissue scaffolds for skeletal muscle cell differentiation would likely be impractical. This dissociation step would add to the cost of production, but these costs could be outweighed by the advantages of some synthetic polymers. Based on current knowledge, it is expected that materials such as poly(methyl vinyl ether‐alt‐maleic anhydride) (PMVE‐alt‐MA) and poly[2‐(methacryloyloxy) ethyl dimethyl‐(3‐sulfopropyl) ammonium hydroxide] (PMEDSAH) fit into this category. In addition, polymers such as PLA, PLGA, PCL, and polyethylene glycol (PEG) can be currently considered for similar applications, and there is reason to believe that it might be possible to establish faster degrading scaffolds to use as tissue embedded structures in certain configurations if degradation products are nonharmful to cells and do not remain in the final CM product. However, this is currently a hypothesis that requires further analysis.

PLGA is versatile and can be used in combination with other materials to produce microcarriers,^[^ ^276^ ^]^ electrospun fibers,^[^ ^199^ ^]^ porous scaffolds,^[^ ^223^ ^]^ hydrogels,^[^ ^229^ ^]^ films,^[^ ^277^, ^278^ ^]^ and 3D printed structures.^[^ ^277^ ^]^ For instance, Kankala et al. have fabricated highly porous spheres composed of a PLGA–gelatin blend that supported adhesion, proliferation, and improved differentiation of C2C12 murine myoblasts.^[^ ^276^ ^]^ In another study, Shin et al. have described the production of nanofibers through electrospinning of PLGA modified with arginylglycylaspartic acid (RGD) motifs, which could support the proliferation and growth of C2C12 myoblasts.^[^ ^199^ ^]^ Chen et al. used 3D printing to fabricate PLGA scaffolds, which supported C2C12 myoblast growth at a faster rate than cells grown in 2D PLGA films and were able to facilitate myotube alignment and differentiation.^[^ ^277^ ^]^

PLA can also form fibers through rotary jet spinning, and rat cardiomyocytes have been shown to spontaneously align on these fibers, supporting the fabrication of beating cardiac muscle tissue.^[^ ^198^ ^]^ Aligned electrospun PLA also supported the adhesion, proliferation, and differentiation of C2C12 myoblasts.^[^ ^197^ ^]^

Successful growth and differentiation of C2C12 myoblasts have been demonstrated in PEG hydrogels.^[^ ^251^ ^]^ In another study, hydrogels composed of a variety of materials, including PEG, were cast around a sacrificial 3D printed vascular‐like network (see Section 6.3) and were able to support perfusion of media through the hydrogel scaffold once the sacrificial material was dissolved.^[^ ^227^ ^]^

High‐porosity surfaces composed of various ratios of PLA and PLGA have been evaluated for their capacity to support the differentiation of mouse myoblasts.^[^ ^223^ ^]^ PLGA alone could not support cell differentiation into elongated myotubes due to inadequate stiffness, but stiffer scaffolds with PLA content ranging from 25% to 100% were able to support myotube formation.^[^ ^223^ ^]^ PLGA can be combined with PEG into the copolymer PEG–PLGA–PEG, which produces a hydrogel that can be seeded with muscle‐derived stem cells to fabricate a biocompatible tissue construct with the ability to support ECM protein deposition.^[^ ^229^ ^]^ Unlike fossil fuel‐derived polyesters, PLA can be synthesized by lactic acid fermentation of sugars, while PLGA is produced through glycolic acid fermentation.^[^ ^279^ ^]^

The aliphatic polyester PCL has gained interest for tissue engineering applications due to its biocompatibility and ability to form a variety of scaffold structures.^[^ ^280^ ^]^ For instance, a bilayer of PCL and anisotropic methacrylated alginate has been shown to support the differentiation of C2C12 myoblasts into aligned myotubes, which could contract following electrical stimulation.^[^ ^209^ ^]^ Semialigned electrospun fibers composed of PCL and gelatin were also shown to support murine L6 myoblast growth and differentiation.^[^ ^206^ ^]^ PCL has also been investigated as a substrate for selective laser sintering, an additive manufacturing technology in which a laser is used to fuse a powdered material (in this case PCL) into a solid 3D structure, which was shown to be effective as a scaffold for MSCs.^[^ ^281^ ^]^

Numerous other synthetic materials have previously been assessed for their potential to support cell anchorage and growth, such as PMVE‐alt‐MA, which has been shown to support the adhesion and proliferation of human ESCs and iPSCs,^[^ ^282^ ^]^ and PMEDSAH, an inexpensive methacrylate derivative that has been used to culture ESCs.^[^ ^230^ ^]^ GelMA (discussed further in Section 5.3) is a semisynthetic material with attractive features for tissue engineering applications including biocompatibility, improved mechanical stability, and slower degradation rates than gelatin.^[^ ^234^ ^]^ Much like gelatin, GelMA retains RGD motifs and therefore is naturally adhesive.^[^ ^283^ ^]^ There is limited information concerning the biodegradability and edibility of PMVE‐alt‐MA and PMEDSAH, and therefore it remains unknown if these materials will be appropriate for CM applications if not removed from the final product. Likewise, GelMA is likely to be less desirable than gelatin for CM applications due to its slower degradation profile.

PLGA and PLA byproducts can be discharged from the human body at low concentrations, though their accumulation can have detrimental effects.^[^ ^279^ ^]^ Lactic acid is a major metabolite of CM and other animal cell production, resulting from the breakdown of glucose in highly proliferative cells.^[^ ^284^ ^]^ While generally considered a waste product because it becomes toxic to cells at sufficiently high concentrations (20 × 10^−3^–40 × 10^−3^ m), the amount of lactic acid produced by the CM industry at scale could be valorized as a sidestream to support PLA production, contributing to a more circular bioeconomy. Lactic acid or lactic acid‐producing bacteria are often added to or present in a variety of foods, including sausages, raw and cooked meat, sourdough, and dairy products.^[^ ^285^, ^286^ ^]^ Lactic acid can be found in feta cheese at concentrations of 18.19 ± 0.27 g kg^−1^ (209.5 × 10^−3^ m, assuming a density of 1037 g L^−1[^ ^38^, ^287^ ^]^) and in cheddar at 18.80 ± 0.03 g kg^−1^ (98.8 × 10^−3^ m, assuming a density of 473 g L^−1[^ ^38^, ^288^ ^]^).^[^ ^289^ ^]^ The main barrier to use of PLGA and PLA as integrated scaffolds is likely to be the presence of incompletely degraded scaffold material in the final product. It is also possible that toxicity of breakdown products to the cells during culture could occur with more rapidly degradable forms of these materials.

PCL can take more than 10 months to fully degrade, although its degradation is mediated by multiple mechanisms and can be accelerated by modifications in pH or through enzymatic dissociation.^[^ ^290^ ^]^ Lipase has been shown to degrade PCL in a dose‐dependent manner.^[^ ^291^ ^]^ Degradation products derived from PCL include hydroxycaproic acid, which is generated after PCL hydrolysis and can enter the Krebs cycle or be discarded through urine and does not appear to accumulate in tissues.^[^ ^292^ ^]^

PEG is approved as a food additive with a maximum allowed level of 1–70 g kg^−1^ of food (or 0.1–7%), depending on the food category,^[^ ^293^ ^]^ though the food categories in which PEG is approved are those typically consumed in small quantities (such as chewing gum or sweeteners), so it remains unclear whether PEG would pose a food safety concern at the concentrations it might be present in a scaffold.

The use of PLGA, PLA, PCL, and PEG in fully degradable scaffolds or those that remain in the product is currently not likely to be feasible. With further innovations, it may be possible to speed up degradation rates of these materials sufficiently that they could be used (see Section 8). Such efforts must take into account the food safety, nutritional, and organoleptic impacts of any remaining scaffold material—including both nondegraded scaffold and breakdown products—in the final CM product as well as the concentrations of breakdown products during the culture period and any resulting impacts on the viability or behavior of the cells.

### 5.2 Self‐Assembling Peptides

Self‐assembling peptides (SAPs) have been investigated for use as scaffolds for tissue engineering and as materials for 3D bioprinting due to their versatility and ability to mimic ECM properties.^[^ ^294^ ^]^

A combination of two SAPs, one containing an RGD adhesion motif (see Section 6.1) and a cleavage site, was used to create peptide coatings designed to control cellular attachment and detachment^[^ ^295^ ^]^ with the goal of enabling continuous cell production without the need for batch harvest. This may be a promising strategy for the proliferation stage of CM production, and this application is being further developed by CellulaREvolution.^[^ ^296^ ^]^

Arab et al. have investigated the application of two SAPs, produced through solid‐phase peptide synthesis by a commercial manufacturer and termed CH‐01 and CH‐02, for their suitability as scaffolds for muscle cells and their ability to serve as bioinks for 3D bioprinting.^[^ ^297^ ^]^ The authors found that these peptide hydrogels possess a fibrous architecture akin to that of bovine collagen type I and that both CH‐01 and CH‐02 allowed adequate alignment of C2C12 myoblasts after eight days of culture, while myoblasts seeded in alginate–gelatin scaffolds were randomly aligned.^[^ ^297^ ^]^

Another SAP with interesting properties for tissue engineering applications is RADA16 (arginine–alanine–aspartic acid–alanine 16, RADARADARADARADA), a short protein sequence composed of 16 amino acid residues in periodical repetitions that can form stable hydrogels with nanofibrous structures when in contact with a saline solution.^[^ ^236^, ^237^ ^]^ RADA16 was shown to support the proliferation and osteogenic differentiation of rabbit dedifferentiated fat cells induced by medium supplementation of osteogenic factors, and these cells secreted calcium‐containing mineralized ECM after 14 days of culture.^[^ ^237^ ^]^ Gao et al. described an RADA16 scaffold with the osteopontin‐derived motif serine–valine–valine–tyrosine–glycine–leucine–arginine (SVVYGLR) that supported seeding of bone marrow‐derived MSCs (BM‐MSCs).^[^ ^238^ ^]^ This scaffold allowed in vivo revascularization of cardiac tissue after myocardial infarction in rats, higher engraftment capacity of BM‐MSCs, and decreased apoptotic events.^[^ ^238^ ^]^ Furthermore, Zhou et al. have incorporated transforming growth factor‐beta (TGF‐*β*) into RADA16 scaffolds seeded with BM‐MSCs, which allowed controlled release of the factor into the cellular environment and improved proliferation capacity.^[^ ^236^ ^]^ Incorporation of costly growth factors into scaffolds can support cost reduction efforts by CM companies by localizing them to where they are needed. Incorporation of different growth factors or differentiation triggers in different regions of the scaffold would also enable spatial heterogeneity when guiding cell fate, thus allowing manufacturers to designate patterning such as meat muscle/fat marbling.

RADA16 and methylcellulose have been used as a bioink to print scaffolds embedded with human or murine MSCs, and the 3D‐printed construct containing murine MSCs was shown to support adipogenic differentiation and lipid accumulation following medium induction.^[^ ^298^ ^]^ SAPs’ application in additive manufacturing has also shown promising results for printing constructs containing fibroblasts and BM‐MSCs.^[^ ^299^ ^]^

Kumada et al. assessed the capacity of two designer SAPs composed of repeating units of amino acids valine–glutamate–valine–lysine, VEVKVEVKV (VEVK9) and VEVKVEVKVEVK (VEVK12), to support the adhesion and growth of fibroblasts upon modification with various adhesion motifs and compare them with nonfunctionalized VEVK9 or VEVK12.^[^ ^239^ ^]^ These peptides could self‐assemble into nanofibrous hydrogel matrices in salted water, and cell attachment and proliferation rates were largely improved in functionalized VEVK9 scaffolds, while VEVK12 was less efficient in allowing fibroblast proliferation even when containing cell adhesion domains.^[^ ^239^ ^]^

The application of SAPs in CM has not yet been addressed in published studies and could be hindered by high manufacturing costs under conventional strategies, such as peptide synthesis. Potential approaches that could be evaluated to reduce the hurdles of SAP production for CM scaffolding include optimizing current techniques and using recombinant organisms.^[^ ^300^ ^]^ Cell‐free systems provide additional opportunities in peptide manufacturing without relying on microbial hosts^[^ ^301^ ^]^ that could be assessed for the production of SAPs.

### 5.3 Extracellular Matrix Molecules

Ultimately, the goal of a scaffold is to mimic relevant properties of the ECM, including its mechanical strength and flexibility, its effects on cell behavior, and—in the case of CM—its nutrient composition. The complexity of the ECM can likely be substituted by simpler scaffolding structures containing one or more of the key structural proteins, growth factors, transcription factors, and cytokines to stimulate normal cell behavior and ECM secretion.^[^ ^275^ ^]^ Although animal‐derived ECM proteins are a poor choice for use in CM, these same components could be produced using microbial fermentation, plant molecular farming,^[^ ^302^ ^]^ or cell‐free systems,^[^ ^301^ ^]^ and mixed in defined ratios, with the formula tailored to the needs of the cell type(s) in question and the desired outcomes. The final CM product will contain both whatever scaffold material is not degraded during the culture period as well as additional ECM components and structures secreted by the cells, in effect bootstrapping some of the complexity of the natural ECM. Beyond its applicability to CM, such ECM substitutes would improve reproducibility in other fields that currently rely on Matrigel.^[^ ^275^ ^]^

The collagens are a large and diverse family of proteins that make up the bulk of the mammalian ECM and have been extensively investigated as a biomaterial. Collagen has several advantages relevant to tissue engineering, including its mechanical strength, its adaptability to different applications based on the type(s) of collagen used and their post‐translational modifications, and its ability to promote adhesion, proliferation, and differentiation of a wide variety of cell types.^[^ ^303^ ^]^ Collagen‐containing scaffolds include microcarriers,^[^ ^169^, ^170^ ^]^ porous scaffolds,^[^ ^248^, ^249^ ^]^ hydrogels,^[^ ^226^, ^240^ ^]^ and films.^[^ ^249^ ^]^ Several bioprinting‐based strategies have also utilized collagen.^[^ ^242^, ^243^, ^244^, ^245^, ^304^ ^]^ In addition, collagen I has been shown to interact with and serve as a slow‐release reservoir for bFGF.^[^ ^248^ ^]^ All collagens share a triple‐helix structure but differ in amino acid sequence and overall organization.^[^ ^305^, ^306^ ^]^ Multiple collagen molecules are often organized into fibrils, especially in connective tissues, though other structures are also common.^[^ ^305^ ^]^ While collagen hydrogels do not inherently feature the sort of well‐ordered structure that would be necessary for mimicking muscle tissue, anisotropy can be introduced simply by clamping the gels along a single axis while letting them freely contract along the other one or two axes.^[^ ^307^ ^]^ This is discussed further in Section 6.2.

Collagen can also be used for scaffolding in its partially hydrolyzed form, i.e., as gelatin or as partially hydrolyzed and methacrylated GelMA. Fibrous scaffolds produced by immersion rotary jet spinning of porcine gelatin have been successfully used to grow rabbit myoblasts and bovine smooth muscle cells, resulting in final products with a reasonably well‐aligned structure, although texture profile analysis revealed some differences from conventional meat.^[^ ^59^ ^]^ Similarly, salmon gelatin was added to scaffolds composed of alginate and agarose with glycerol as a plasticizer, and the resulting hydrogel was freeze‐dried to create a porous scaffold.^[^ ^122^ ^]^ The resulting scaffold exhibited the mechanical properties of the plant‐based components and the cell adhesive properties of the gelatin and thereby was able to support adhesion, viability, and proliferation of C2C12 myoblasts over 72 h of culture.^[^ ^122^ ^]^ Gelatin has also been successfully used as a scaffold for engineered adipose tissue, with pores and channels both formed by dissolving sacrificial structures constructed from alginate.^[^ ^227^ ^]^ Costantini et al. have shown that GelMA hydrogels can support the proliferation of C2C12 myoblasts and differentiation into myotubes.^[^ ^235^ ^]^ GelMA hydrogels composed of fish gelatin have also been reported to provide an adequate environment for NIH3T3 embryonic fibroblasts, supporting the adhesion and proliferation of these cells for five days.^[^ ^234^ ^]^ Patterned GelMA fibers obtained using micromolding have been shown to sustain C2C12 myoblast viability similarly to unpatterned fibers, though myoblast alignment is significantly improved on patterned GelMA fibers.^[^ ^202^ ^]^ Cells grown on patterned GelMA fibers exhibited increased expression of myogenic markers of myotube formation, including myosin heavy chain (MHC) and sarcomeric actin.^[^ ^202^ ^]^ GelMA has also been used as a bioink component, with C2C12 myoblasts in the printed constructs capable of surviving over several weeks and differentiating.^[^ ^187^ ^]^ Because gelatin and GelMA share many of the same advantages, and the slower degradation rate of GelMA could be a disadvantage rather than an advantage in the context of CM, animal‐free forms of gelatin are likely to be preferable to GelMA for use in CM scaffolds.

While tissue engineering research has largely relied on animal‐derived collagen, animal‐free collagens have been successfully produced in plants, bacteria, and yeast,^[^ ^81^ ^]^ opening the door for collagen or gelatin as a viable scaffold material for CM. Certain bacterial species express proteins with collagen‐like sequences and properties. A collagen‐like protein expressed by *Streptococcus pyogenes* showed good compatibility with cultured human and murine cells.^[^ ^308^ ^]^ Whereas mammalian collagens contain hydroxyproline and would therefore need to be coexpressed with enzymes for post‐translational modification if expressed in recombinant systems, these bacterial collagen‐like sequences do not suffer from this limitation. Furthermore, bacterial collagen‐like proteins may be functionalized by the addition of binding motifs from mammalian ECM proteins to tune their adhesive properties toward specific cell types.^[^ ^309^, ^310^ ^]^ By introducing a silk consensus sequence into the gene for the bacterial collagen‐like protein Scl2, hybrid Scl2‐silk scaffolds were produced that, when modified with fibronectin‐binding sites, could support the proliferation of human MSCs to a similar degree as collagen I.^[^ ^311^ ^]^ Integrin binding sites were also effective at supporting cell adhesion and proliferation, though to a lesser extent.

A recent study found that collagen was less able to support the formation of contractile muscle tissue as well as less effective at promoting myotube maturation, as assessed by *α*‐actinin staining, compared to scaffolds composed of a mixture of fibrin and Matrigel.^[^ ^119^ ^]^ Hydrogels of fibrin alone have been investigated for use as CM scaffolds. Like collagen, fibrin can support the growth of constructs with visibly aligned myotubes when anchored between two fixed points.^[^ ^115^ ^]^ Fibrin has also been used as a bioink component for CM,^[^ ^121^ ^]^ and in another study, cell‐laden fibrin gels were cast around 3D printed sacrificial channels to create perfusable constructs.^[^ ^228^ ^]^

Laminins, a major component of the basal lamina, have also been investigated for their potential to support cell attachment, growth, and differentiation, with certain laminin isoforms showing particular potential. Due to the differences in function between laminin isoforms and the difficulty of producing pure laminins from animal or animal cell culture sources, methods for recombinant laminin production have been developed.^[^ ^312^ ^]^ Human ESCs proliferated more efficiently in agitated culture on microcarriers coated with positively‐charged poly‐l‐lysine and either vitronectin or laminin, relative to those coated with poly‐l‐lysine, vitronectin, or laminin alone,^[^ ^313^ ^]^ suggesting that the function of laminins in supporting cell adhesion and proliferation may be enhanced by combining them with other compounds.

Laminins are trimers with *α*, *β*, and *γ* subunits and are often named according to their constituent chains. For example, laminin 421 refers to the laminin composed of *α*4*β*2*γ*1 subunits.^[^ ^314^ ^]^ Compared to gelatin, Matrigel, and a variety of laminin isoforms including the commonly used laminin 111, laminin 521 showed the most consistent ability to support human and mouse myoblast proliferation and differentiation, including differentiation following multiple passages.^[^ ^315^ ^]^ While not investigated to the same extent as laminin 521, laminin 511 was effective at supporting short‐term proliferation and differentiation of mouse satellite cells.^[^ ^315^ ^]^ In another study, laminin 511 was able to support the long‐term proliferation of mouse ESCs and expression of pluripotency markers, whereas laminins 111, 332, and 411, Matrigel, gelatin, and poly‐d‐lysine led to either premature differentiation or cell death.^[^ ^316^ ^]^ These results together point to the laminins containing the *α*5 chain as promising scaffolds for muscle tissue engineering, including for CM. Specific laminin isoforms are associated with different states of the muscle stem cell niche. Changes are observed between embryonic and adult muscle as well as between regenerative and dystrophic states, and the primarily embryonic *α*1 and *α*5 chains are upregulated during regeneration.^[^ ^317^ ^]^ In addition to some laminins being more amenable to stem cell proliferation, it may be the case that the best laminin isoform for use in a CM scaffold will depend on the origin of the cells or the desired end product. Caution is also warranted when translating findings from mouse studies to agriculturally‐relevant species since there might be important differences in the utilization of some isoforms. Interestingly, long‐term culture on various laminins or Matrigel led to differences in expression of integrins, suggesting a possible mechanism by which the substrate might influence cells’ later propensity for adhesion, proliferation, and differentiation under different culture conditions.^[^ ^315^ ^]^

A combination of gelatin and fibronectin has been used in the “cell accumulation technique” in which cells are coated in a thin layer of ECM proteins and then self‐assembled into a tissue‐like structure, obviating the need for a preassembled scaffold, at least in applications where only a thin construct is required.^[^ ^318^, ^319^ ^]^ This method allowed for the creation of heterogeneous constructs made up of multiple distinct layers of different cell types or labeled cells and furthermore led to the development of fine capillary‐like structures when a layer of endothelial cells was included in the construct.^[^ ^319^ ^]^ In a later study, the technique was successfully applied to C2C12 myoblasts.^[^ ^318^ ^]^ Differentiation was initially impaired in thick constructs, but this was rescued by the application of a Rho kinase inhibitor to the extent that coated and inhibitor‐treated cells formed tissue constructs that were both thicker and more fully differentiated than noncoated controls.^[^ ^318^ ^]^

The ECM protein vitronectin has shown some promise as a substrate for growth of human ESCs in 2D culture as well as on microcarriers^[^ ^320^ ^]^ and, as mentioned above for laminin, showed better performance when combined with positively charged poly‐l‐lysine.^[^ ^313^ ^]^ Vitronectin surfaces patterned using photolithography were capable of supporting the attachment of C2C12 myoblasts and fusion into myotubes.^[^ ^321^ ^]^ Interestingly, when PGA, PLGA, or collagen‐based scaffolds were incubated in serum‐containing medium, vitronectin and, to a lesser extent, fibronectin from the serum adsorbed onto the scaffolds and mediated adhesion of smooth muscle cells via interaction with integrin receptors.^[^ ^249^ ^]^ This suggests that recombinant vitronectin and/or fibronectin, either added directly to the scaffold or included in the culture media, might be effective in maintaining strong adhesion of cells to scaffolds under serum‐free conditions.

### 5.4 Plant and Fungus‐Derived Materials

Promising approaches for scaffolding materials also include those derived from plants or fungi. The use of plant‐based proteins as biomedical tissue engineering scaffolds has been thoroughly reviewed elsewhere.^[^ ^322^ ^]^ In some cases, it is possible to take advantage of the natural 3D structure of such materials, such as by decellularization of plant tissues. Plant tissues may be decellularized by detergent‐based (SDS followed by Triton X‐1000) or detergent‐free (heated bleach solution) methods, with some optimization needed to adapt each method to a new species.^[^ ^323^ ^]^ Because plant tissues naturally feature vasculature and porous structures, the use of decellularized plants may facilitate oxygen and nutrient transport. Plant materials that have undergone some minimal processing steps, including those commonly used in the food industry, may also provide useful structural features. More extensive purification and processing using methods such as electrospinning, which is commonly used for biomedical applications, may also be applied to plant‐ and fungus‐derived materials.

Spinach has been investigated for its potential as a scaffold due to its wide availability, dense vascularization, and wide petiole (the stalk attaching the leaf to the stem).^[^ ^32^, ^118^, ^324^ ^]^ Spinach leaves have been tested for their utility as scaffolds for CM and were able to support bovine satellite cell survival over a 14‐day culture period, differentiation of some cells, and strong directional alignment in some samples, all without functionalization^[^ ^118^ ^]^ (Figure 5b; functionalization is discussed in Section 6.1). Spinach leaves may be decellularized either by introducing the decellularization reagents through a cannula or by immersion. The latter is preferred for larger‐scale applications due to the time required for individual cannulation of spinach leaves.^[^ ^118^ ^]^ Decellularized spinach leaves were shown to support the perfusion of dyes, media, and cells through their vasculature as well as the attachment of endothelial cells within the vasculature and of MSCs and cardiomyocytes on the surface of the leaves without functionalization.^[^ ^32^ ^]^ In another study, spinach leaves functionalized with collagen IV or fibronectin were compared to uncoated leaves, with largely similar outcomes regarding sarcomere length, maximum contractile strain, and average cell numbers.^[^ ^324^ ^]^ While the constructs studied here were limited to the size of a spinach leaf (or a portion thereof), the presence of an intact vasculature raises the possibility that decellularized leaves could be incorporated into a larger perfusable construct.

Campuzano et al. identified celery as a promising candidate for use as a decellularized plant scaffold based on its strong anisotropy and the presence of optimally sized pores for promoting myoblast alignment.^[^ ^125^ ^]^ Indeed, C2C12 myoblasts adhered well to decellularized celery and showed strong alignment in the direction of the vascular bundles.^[^ ^125^ ^]^ This was achieved without biofunctionalization of the scaffold, which the authors attribute to the physical cues provided by the highly anisotropic plant tissue or the presence of adhesive proteins in the FBS, but it was not ruled out that biofunctionalization would have improved the performance of decellularized celery as a scaffold.^[^ ^125^ ^]^ Similar results have recently been reported using both decellularized green onion bulbs^[^ ^325^ ^]^ and decellularized grass.^[^ ^131^ ^]^

Decellularized apples have been shown to support attachment and survival of C2C12 myoblasts over a culture period of two weeks;^[^ ^240^ ^]^ attachment and proliferation of iPSCs and differentiation into bone tissue;^[^ ^326^ ^]^ and adhesion, proliferation, and differentiation of preadipocytes.^[^ ^327^ ^]^ In addition, apple‐based scaffolds could be combined with temporary or permanent hydrogels derived from gelatin or collagen, which was suggested as a strategy for increasing initial cell contact with the scaffold by increasing the viscosity of the solution and for delivery of biochemical cues to the construct.^[^ ^240^ ^]^

Fontana et al. tested a wide array of decellularized plant species for their compatibility with human cells.^[^ ^328^ ^]^ MSCs and dermal fibroblasts grew well on decellularized parsley stems that were either mineralized or functionalized with an ECM adhesion motif, whereas other plant tissues supported short‐term growth only.^[^ ^328^ ^]^ Orchid pseudobulbs supported long‐term growth for fibroblasts but not MSCs, suggesting some level of species and cell type specificity.^[^ ^328^ ^]^ *Anthurium magnificum*‐based scaffolds also supported the attachment of endothelial cells, although long‐term culture was not assessed.^[^ ^328^ ^]^ Fibroblasts showed a tendency to orient themselves according to the topography of the plant scaffold, with the strength of this effect varying according to scaffold species.^[^ ^328^ ^]^ For the purposes of designing scaffolds for CM, there is a great deal that can be learned from such studies on cells from human, mouse, and other species. However, additional research is required to test the applicability of these scaffolds in agriculturally relevant species and optimize their properties for compatibility with the desired cells.

Besides plants, certain species of bacteria and algae also produce cellulose. Bacterial cellulose‐based scaffolds have been evaluated for biomedical tissue engineering applications^[^ ^157^ ^]^ and could also potentially be used as scaffolds for CM. The use of fermented bacterial nanocellulose as scaffolding for CM is being addressed by Cass Materials, based on promising early tests indicating that muscle cells could adhere to the highly porous scaffolds and form fibers.^[^ ^329^ ^]^ The company is developing large (up to several cm thick) scaffolds as well as porous microcarriers tailored to the CM industry. In addition, use of decellularized green algae as tissue engineering scaffolds was recently demonstrated.^[^ ^220^ ^]^ Most of the cellulose‐based scaffolds described above exhibited a porous structure (Figure S1, Supporting Information), with the exception of the green algae *Cladophora*, which was primarily fibrous.^[^ ^220^ ^]^

A key consideration for the use of cellulose‐based scaffolds, whether in the form of decellularized plants or otherwise, will be the effects of the scaffold on the organoleptic and nutritional properties of the final product. Bacterial cellulose‐based scaffolds are generally assumed by biomedical tissue engineers to be nondegradable due to the lack of cellulase in humans and other mammals; consistent with this, degradation of bacterial cellulose scaffolds implanted into rats was minimal over 16 weeks.^[^ ^157^ ^]^ Scaffolds made from decellularized apple degraded substantially over eight weeks when implanted into mice, yet scaffold material remained at the end of this period.^[^ ^158^ ^]^ Therefore, it can be assumed that complete degradation of cellulose scaffolds is unlikely under anticipated CM culture conditions, even with a long differentiation phase. Whether this is an advantage or a disadvantage remains to be determined. Enhanced fiber content due to remaining scaffold material could be seen as an advantage from a nutritional perspective, but the suitability of such products from an organoleptic perspective will need to be carefully assessed. Bacterial cellulose scaffolds formed using different methods showed different degrees of degradation,^[^ ^157^ ^]^ and decellularized apple‐based scaffolds degraded more quickly^[^ ^158^ ^]^ than those formulated from bacterial cellulose (though this might be partially attributable to different culture conditions), suggesting that some cellulose‐based scaffolds may be more prone to degradation than others. Therefore, if the sensory effects of cellulose scaffolds on the final meat product are undesirable, it may be possible to select cellulose‐based scaffolds capable of mostly or completely degrading over the course of the cultivation period.

Existing methods for processing plant material into human food yield porous structures that may be conducive to cell growth. As discussed in Section 4.2, both textured soy protein^[^ ^123^ ^]^ and soda bread^[^ ^191^ ^]^ have been investigated as scaffolds for CM, with promising early results. Plant‐derived materials have also been investigated for tissue engineering applications in more processed forms. For example, rotary jet spun fibers formed from a combination of cellulose acetate and soy protein hydrolysate were able to support the adhesion and proliferation of human fibroblasts.^[^ ^210^ ^]^

Alginate, a polysaccharide derived from brown algae, may also be a promising scaffolding material for CM. Alginate‐derived tubes have been shown to support high cell density and growth rates of pluripotent stem cells and to be compatible with differentiation protocols^[^ ^231^, ^232^ ^]^ and furthermore have been used to culture bovine adipogenic precursors.^[^ ^233^ ^]^ Alginate has also shown promise as a bioink for 3D printing, including for tissue‐engineered constructs containing adipocytes^[^ ^222^ ^]^ and C2C12 myoblasts.^[^ ^187^ ^]^ Alginate may also be useful for supporting large‐scale proliferation; for example, alginate beads have been tested as microcarriers^[^ ^330^ ^]^ and as microspheres in which cells may be encapsulated.^[^ ^331^ ^]^ Because alginate hydrogels depend on the presence of calcium ions, the gelation process can be reversed simply by the addition of a calcium chelator, a property that Contessi Negrini et al. took advantage of to create pores and channels from sacrificial alginate‐based structures in a gelatin scaffold.^[^ ^227^ ^]^ Although alginate suffers from certain limitations such as a lack of cell adhesiveness when unmodified,^[^ ^272^ ^]^ other properties such as its reversible gelation,^[^ ^227^ ^]^ tunable mechanical properties,^[^ ^222^, ^331^ ^]^ and ease of being formed into different geometries^[^ ^187^, ^231^, ^331^ ^]^ make it a potentially promising candidate, especially if combined with other materials with complementary properties. The use of partially oxidized alginate may be a viable strategy to accelerate its degradation.^[^ ^331^ ^]^ The tendency of alginate to form soft hydrogels^[^ ^222^, ^272^ ^]^ makes it particularly suited for use with adipogenic cells, though it is possible by changing the calcium concentration to produce alginate hydrogels with sufficient stiffness to support myogenic differentiation as well.^[^ ^331^ ^]^

Like plants, certain fungal species have structural features that might make them desirable as scaffolds for CM.^[^ ^152^ ^]^ In fact, the startup Excell, which was spun out of sustainable biomaterials company Ecovative Design, produces fungal mycelium‐based scaffolds for use in the CM industry, and Ecovative Design has filed a patent for the use of mycelial scaffolds for both CM and biomedical applications.^[^ ^152^ ^]^ The air pores formed by *Armillaria luteobubalina* have been shown to conduct oxygen^[^ ^332^ ^]^ and could serve the same function in the context of a scaffold. Freeze‐dried mycelial mats derived from *Aspergillus* have been shown to support adhesion and proliferation of human keratinocytes with superior performance compared to 2D culture.^[^ ^219^ ^]^ Fungal hemolysins present in the scaffold led to significant hemolysis of red blood cells but could be removed by treatment with *β*‐mercaptoethanol.^[^ ^219^ ^]^ Mycelia have also been identified as a promising category of candidates for use in wound care due to their similarities to native mammalian ECM and favorable oxygen transport properties,^[^ ^333^ ^]^ both of which are important features for CM scaffolds.

Chitosan is a polymer found in the skeletons and shells of insects and crustaceans and is also produced by certain fungi. Directional freezing of crustacean‐derived chitosan forms a structure featuring elongated pores capable of supporting adherence and differentiation of C2C12 myoblasts, with the final myotube diameter dependent on the initial chitosan concentration.^[^ ^274^ ^]^ Fungal chitosan sponges created by a similar directional freezing technique were able to support adhesion of *Drosophila* muscle progenitors as well as differentiation of a small percentage of cells, and in some cases, muscle fibers were aligned to the pores of the scaffold.^[^ ^334^ ^]^ Membranes composed of one part chitosan to two parts of the plant‐derived polysaccharide pectin supported adhesion and proliferation of human adipose‐derived stem cells, whereas those made from a five‐to‐one ratio showed little to no growth over the 7‐day culture period.^[^ ^247^ ^]^ Because chitosan supports cell adhesion without the need for functionalization,^[^ ^274^, ^334^ ^]^ can form scaffolds with a range of mechanical properties compatible with those needed for the growth of myofibers and other meat‐relevant cells (reported Young's moduli of 4–125 kPa^[^ ^274^ ^]^ and 2–5 kPa^[^ ^334^ ^]^), and is already used in edible coatings in the food industry,^[^ ^335^ ^]^ it should be considered a promising material for CM scaffolding and worthy of further research. Fungal‐derived scaffolds might also have beneficial effects beyond their role as a support structure. For example, certain polysaccharide fractions from *Grifola frondosa* were shown to stimulate both proliferation and collagen synthesis when added to cultured mouse fibroblasts.^[^ ^336^ ^]^ In addition, cultures of *Lentinus edodes* have been shown to produce a heat‐stable compound with antibacterial activity.^[^ ^337^ ^]^

## 6 Engineering Biological and Structural Complexity

While large‐scale cultivation of mammalian cells is routinely performed in the biopharmaceutical and cell therapeutic industries, CM faces the additional challenge of producing a complex, intact tissue as the final product rather than a cell slurry or a secreted product. This section will discuss specific considerations that arise as a result of this focus on large, complex tissues.

### 6.1 Cell Adhesion

Some scaffold materials are inherently functional and do not require further modification for cell attachment, including silk fibroin,^[^ ^338^ ^]^ textured vegetable proteins,^[^ ^123^ ^]^ and gelatin.^[^ ^59^ ^]^ By contrast, other materials’ ability to support cell adhesion can be improved through biochemical enhancements, such as for plant‐sourced materials such as alginate^[^ ^272^ ^]^ and synthetic polymers like PCL^[^ ^209^, ^280^ ^]^ and PLGA.^[^ ^199^, ^278^ ^]^ Since these materials possess a suite of additional benefits that make them promising candidates for CM applications, such as cell compatibility and desirable mechanical properties, improving cell anchorage is essential to ensure their applicability as scaffold components. A variety of strategies have been described that could improve cell adhesion to such surfaces, many of which are based on functionalization with adhesion motifs identified in ECM proteins. The following sections will discuss promising adhesion motifs, cost and regulatory considerations related to the use of such motifs, and nonpeptide‐based strategies for improving adhesion.

#### 6.1.1 RGD Motifs

Functionalizing scaffolds is usually performed by cross‐linking a material with molecular moieties that cell membrane‐associated proteins can recognize and bind to. One such alteration is the addition of arginyl–glycyl–aspartic acid (RGD) motifs. This tripeptide is found in ECM proteins such as fibronectin and is a major binding site for transmembrane integrins.^[^ ^339^ ^]^ Accordingly, RGD peptides can be added to scaffolds to promote the adhesion of multiple cell types relevant for CM applications. Approaches for scaffold functionalization with RGD may vary according to the type of scaffold and include using carbodiimide chemistry^[^ ^272^, ^340^ ^]^ or mixing functional motifs with the scaffold materials.^[^ ^239^ ^]^ There should be careful consideration of scaffold functionalization methods, as some chemical conjugation techniques could potentially introduce harsh reagents or chemical modifications that should be assessed from a food safety standpoint.

Alginate is an example of a scaffold material that can be modified with RGD motifs, and it has been shown that alginate hydrogels containing RGD can facilitate the adhesion of C2C12 myoblasts to the scaffold.^[^ ^340^ ^]^ In this report, an increase in RGD density was associated with higher cell proliferation and differentiation rates. In addition, modifying PLGA scaffolds with RGD has allowed significant improvements in C2C12 myoblast cell area as well as myoblast fusion and myotube maturation index.^[^ ^199^ ^]^ In this study, staining of late differentiation marker MHC was only apparent in RGD‐containing scaffolds, and the addition of graphene oxide further improved myogenic differentiation capacity. RGD motifs have also been incorporated into SAPs to enhance cell adhesion.^[^ ^295^ ^]^ Moreover, research from Chandler et al. using 3T3‐L1 preadipocytes revealed an increase in average cell number and total cell area in RGD‐modified alginate scaffolds compared to controls.^[^ ^272^ ^]^

#### 6.1.2 Cellulose‐Binding Domains

Adhesion motifs such as RGD can also be combined with cellulose‐binding domains (CBDs) to increase the functionality of certain scaffold materials. For instance, CBD–RGD complexes can improve the attachment and viability of human microvascular endothelial cells and murine embryonic fibroblasts when added to bacterial cellulose scaffolds.^[^ ^341^, ^342^ ^]^ Similar improvements in functionality have been observed when adding CBD–RGD to alginate scaffolds, which promote the chondrogenesis of MSCs.^[^ ^343^ ^]^ CBDs can be found in plant enzymatic complexes that take part in cellulose degradation and in exposing the catalytic motifs of cellulosic substrates.^[^ ^344^ ^]^ Therefore, CBDs could be investigated for their application in improving the functionalization of plant scaffolds by combining these domains with mammalian cell adhesion motifs.

#### 6.1.3 PHSRN Motifs

Other peptide moieties have also been shown to improve the adhesive properties of scaffold materials, such as proline–histidine–serine–arginine–asparagine sequences (PHSRN), which are also present in fibronectin. A recent report demonstrated that pluripotent stem cells (iPSCs and ESCs) exhibit higher adhesion rates to culture plates with PHSRN and RGD‐containing (GRGDSP) motifs combined than to plates coated with only one of these motifs or with Matrigel.^[^ ^345^ ^]^ For cultures of embryonic stem cell‐derived MSCs, the addition of PHSRN domains to culture plates coated with fibronectin–gelatin mixtures increased cell proliferation compared with coatings comprised of fibronectin, gelatin, or a combination of both.^[^ ^346^ ^]^ The authors of this study also reported substantially lower cell clustering in fibronectin–gelatin plates with PHSRN motifs after seven days of culture.

#### 6.1.4 GFOGER Motifs

Another peptide sequence that can be used to improve cell adhesion to scaffolds is glycine–phenylalanine–hydroxyproline–glycine–glutamic acid–arginine (GFOGER). This motif is present in collagen molecules and targets integrins such as *α*2*β*1 (CD49b). Adding GFOGER peptides to hydrogels has been shown to improve MSC adhesion with similar strength as RGD motifs.^[^ ^347^ ^]^

#### 6.1.5 IKVAV and YIGSR Motifs

Modifying scaffolds with laminin‐derived domains is another promising strategy for improving the cell‐adhesive properties of certain materials. For instance, adding the isoleucine–lysine–valine–alanine–valine sequence (IKVAV) to 3% hyaluronic acid hydrogels seeded with mouse myogenic progenitors has been shown to promote cell adhesion and spreading throughout the scaffold as well as upregulation of myogenic factors MyoD1 and Pax7.^[^ ^348^ ^]^ Modifying peptide‐based scaffolds with IKVAV appears to be particularly useful for adequate fibroblast attachment.^[^ ^239^ ^]^ In this report, a similar improvement in fibroblast adhesion to scaffolds based on self‐assembling peptides was observed upon modification with tyrosine–isoleucine–glycine–serine–arginine (YIGSR), which is another laminin‐derived cell adhesion domain. Furthermore, combining the proline–valine–glycine–leucine–isoleucine–glycine (PVGLIG) sequence—a cleavage site of MMP‐2—with two RGD motifs was reported to potentiate fibroblast migration through the scaffold.^[^ ^239^ ^]^ Grooved PLGA scaffolds containing YIGSR or RGD peptides grafted with poly‐l‐lysine have also been reported to improve C2C12 myoblast proliferation and differentiation compared to PLGA surfaces devoid of biochemical cues.^[^ ^278^ ^]^

#### 6.1.6 Cost and Regulatory Considerations for Adhesion Motifs

Overall, functionalizing materials with the right ECM motifs allows the adhesion of a plurality of cell types relevant for CM applications to materials that otherwise do not allow adequate cell anchorage. However, peptide synthesis could increase production costs, thereby hindering the application of cell adhesion motifs in CM scaffolds. Therefore, the cost‐efficiency, scalability, and efficacy of adding adhesion domains to scaffolds should be further analyzed for application in CM.

It is noteworthy that the use of RGD peptides in food products is yet to be approved.^[^ ^44^ ^]^ Therefore, companies and researchers should aim to select food‐grade materials that already contain one or more of these adhesion‐promoting peptide sequences when selecting scaffold material candidates to minimize the potential for regulatory hurdles. One approach for including cell adhesion motifs in scaffolds was described in Modern Meadow's patent, where cardosin A was cross‐linked with pectin because cardosin A contains RGD domains.^[^ ^140^ ^]^ Cardosin A is an aspartic protease that can be extracted from the cardoon *Cynara cardunculus* and has been used for cheese‐making due to its milk clotting properties.^[^ ^349^ ^]^ Therefore, investigating plant‐derived peptides already used for food applications that contain amino acid sequences analogous to vertebrate and mammalian cell adhesion domains could be a cost‐effective approach to include these motifs in scaffolds and improve their functionality.

#### 6.1.7 Nonpeptide‐Based Methods

Cellivate Technologies has developed a novel cell adhesion chemistry for tissue culture, glass, and microcarrier coatings composed of various combinations of metal oxides with ≈5–100 nm coating thickness.^[^ ^350^, ^351^, ^352^ ^]^ Because the metal oxides are nonedible, scaffolds containing these coatings can be used only as temporary supports during the proliferation phase and will not be present in the final CM product. The stiffness, roughness, and wettability of the culture surface can also influence cell adhesion,^[^ ^353^, ^354^ ^]^ providing additional opportunities for CM producers to tune the adhesive properties of their scaffolding materials.

### 6.2 Maturation and Alignment

Muscle fibers in vivo are typically found in a regularly aligned pattern, and this alignment contributes to the distinctive textural properties of meat.^[^ ^29^ ^]^ In addition to the direct effects of fiber alignment on perceptions of texture, alignment also serves as a cue that influences muscle cell differentiation, maturation, and gene expression,^[^ ^126^, ^197^, ^200^, ^208^, ^241^ ^]^ though some studies have reported successful alignment of cells without increases in differentiation or strong differentiation without alignment.^[^ ^252^, ^355^ ^]^ Fully mature muscle fibers with the proper amounts of actin, myosin, and myoglobin will be necessary to fully recapitulate the flavor, texture, color, and nutritional content of conventional meat.^[^ ^28^, ^115^ ^]^

Alignment of cells on a scaffold can be confirmed by measuring the angle of orientation of each cell and reporting the percentage of cells oriented within a certain number of degrees relative to either the average alignment or the axis of alignment of the scaffold,^[^ ^115^, ^197^, ^208^, ^252^ ^]^ but is also often simply confirmed visually. Differentiation and maturation may be assessed or quantified using a wide variety of metrics, including cellular aspect ratio,^[^ ^200^ ^]^ percentage of myotubes meeting a certain threshold for the number of nuclei contained,^[^ ^197^ ^]^ myotube length or diameter,^[^ ^208^, ^252^, ^274^ ^]^ or presence of marker genes. Commonly measured markers include myoD,^[^ ^252^ ^]^ myosin heavy chain,^[^ ^126^, ^241^, ^252^ ^]^ alpha‐sarcomeric actin,^[^ ^241^ ^]^ laminin,^[^ ^241^ ^]^ desmin,^[^ ^126^ ^]^ or myogenin,^[^ ^126^, ^252^ ^]^ which may be assessed according to expression level, area, or striation pattern.

Researchers have successfully induced muscle fiber alignment using a variety of strategies, including the use of anisotropic 3D scaffolds or 2D substrates, controlling the position of cells at the start of the culture period using additive manufacturing or acoustic cues, and the use of in vivo‐like cues such as mechanical stretch or electrical stimulation. These methods will be discussed in detail in the following sections.

#### 6.2.1 Anisotropic Biomaterials

Muscle cells can be induced to align by the use of anisotropic biomaterials. For example, as discussed above, electrospinning of a variety of materials can be used to produce aligned fibers that promote unidirectional alignment of muscle cells.^[^ ^59^, ^197^, ^200^, ^205^, ^208^ ^]^ A comparison of human muscle cells cultured on aligned versus randomly oriented electrospun fibers revealed that myotubes in the aligned condition not only mirrored the alignment of the scaffold but were also twice as long after seven days in differentiation media.^[^ ^208^ ^]^ The length of the scaffold fibers is also important; another study reported that short (≈20 µm) fibers promoted the formation of spherical aggregates, whereas longer fibers were able to produce well‐aligned tissues, especially if the scaffold fibers were chemically cross‐linked.^[^ ^59^ ^]^ Anisotropy can also be introduced into materials by directional freezing.^[^ ^334^, ^356^ ^]^ Certain plant tissues—such as parsley stems, celery, green onion bulbs, spinach leaves, and grass—have intrinsically anisotropic features and can induce alignment of cultured muscle cells when used as decellularized scaffolds.^[^ ^118^, ^125^, ^131^, ^325^, ^328^ ^]^ Hollow microfibers produced using microfluidic methods have also successfully been used to culture myocytes and cardiomyocytes and provide the necessary cues to induce cellular alignment along the long axis of the tube.^[^ ^357^ ^]^

#### 6.2.2 Curved, Grooved, or Patterned Substrates

The curvature of the substrate alone may be sufficient for inducing cell alignment and differentiation. Human corneal stromal cells cultured on curved surfaces demonstrated increased expression of ECM‐related genes compared to those grown on planar surfaces.^[^ ^358^ ^]^ Similarly, C2C12 myoblasts expressed more undifferentiated cell markers when grown on planar substrates or on half‐cylinders with diameters above 15–35 mm and more markers of early and late‐stage myogenic differentiation when the substrate diameter was below 10–20 mm, as well as more consistent alignment on substrates with small diameter curvatures.^[^ ^359^ ^]^

Lithography, embossing, and micromolding have also been used to generate anisotropy within tissues. Anisotropy was successfully produced in thin films by casting a biopolymer solution using a laser‐etched mold with appropriately‐sized ridges, leading to better tissue organization and higher expression of muscle differentiation‐associated markers compared to isotropic controls when these films were seeded with myoblasts.^[^ ^126^ ^]^ PEG gels with deep ridges were produced by casting the monomer solution in patterned molds and were shown to support the alignment and differentiation of C2C12 myoblasts.^[^ ^252^ ^]^ A similar approach using ridged modules seeded with bovine myocytes was able to successfully produce an 8 × 10 × 7 mm piece of cultivated “steak” tissue, which showed somewhat similar mechanical properties to a piece of conventional beef tenderloin^[^ ^119^ ^]^ (Figure 5d). Surfaces embossed with grooves have also been demonstrated as a method for inducing alignment of both C2C12 and primary myoblasts.^[^ ^355^ ^]^ Molnar et al. used photolithography to pattern vitronectin into stripes of varying widths and found that 30 µm wide stripes were ideal for inducing C2C12 myoblasts to form isolated myotubes, while 50 µm stripes led to the formation of multiple myotubes on a single stripe, and those 20 µm or thinner were ineffective at inducing alignment and differentiation.^[^ ^321^ ^]^

#### 6.2.3 Introducing Anisotropy through Bioprinting

Bioprinting (see Section 4.5) can also be used to introduce anisotropy.^[^ ^187^ ^]^ For example, tissue‐engineered muscle constructs have been printed using alternating stripes of fibrinogen‐based bioink containing muscle progenitor cells and sacrificial gelatin to induce alignment and provide channels for oxygen and nutrient transport.^[^ ^34^, ^241^ ^]^ Printed constructs of 15 mm^3^ were able to survive for at least six days in vitro and eight weeks in vivo and showed strong alignment of muscle fibers and expression of muscle‐specific markers, whereas nonprinted control constructs containing the same hydrogels and cells showed lower survival, little to no alignment, and poor expression of muscle markers.^[^ ^241^ ^]^ Chaotic bioprinting, in which a static mixer is used to partially mix two inks as they are about to be printed, has been demonstrated to generate alternating regions with and without cells with a combination of C2C12‐laden GelMA‐alginate bioink and pure alginate ink, effectively increasing the resolution of the construct within a single extruded fiber^[^ ^187^ ^]^ (Figure 5e).

#### 6.2.4 Acoustic Cues

As an alternative to bioprinting, cells can be patterned using acoustic cues.^[^ ^360^ ^]^ Mimix Bio aims to apply this method to pattern CM tissues using “sound‐induced morphogenesis.”^[^ ^361^, ^362^ ^]^ C2C12 myoblasts exposed to a 30 s ultrasound standing wave stimulus formed orderly rows, which were maintained over four days of culture and led to an increase in MRF4 expression relative to control conditions.^[^ ^363^ ^]^ A similar method has also been successfully applied in 3D conditions, with a 15 min acoustic stimulus applied to endothelial cells in a collagen solution during the gelation period.^[^ ^364^ ^]^ Whereas cells can be patterned into stripes using simple waveforms, more complex patterns have also been achieved using acoustic hologram‐based techniques.^[^ ^365^ ^]^

#### 6.2.5 Passive, Gradual, or Phasic Stretch

Cellular alignment can be induced by mechanical stimulation as well. This stimulation can take the form of a passive stretch stimulus, for example by constraining cell‐laden collagen gels in one direction while allowing them to freely contract in the other.^[^ ^307^, ^366^, ^367^, ^368^, ^369^ ^]^ A recently described “tendon‐gel integrated printing” method used a combination of bioprinting and passive stretch.^[^ ^121^ ^]^ In this method, thin fibers of cell‐laden gelatin were printed with artificial “tendons” composed of collagen capping each fiber. These “tendons” were used to anchor the constructs as they differentiated and matured, after which multiple fibers bearing different cell types were combined to create CM.^[^ ^121^ ^]^ Two other recent studies also used passive stretch to induce alignment in small‐scale, anchored CM constructs.^[^ ^115^, ^119^ ^]^ Similarly, constraining a ring‐shaped collagen gel around a central post led to strain as the gel contracted, leading the cells within the gel to become aligned.^[^ ^370^ ^]^ Gradual stretching of collagen‐coated membranes seeded with primary cells from avian muscle induced alignment of myotubes and increased the final length of the myotubes by a factor of 2–4.^[^ ^371^ ^]^ Interestingly, this effect was also seen when the membranes were prestretched before adding the cells, which the authors speculated might have been a result of either collagen becoming oriented on a small scale as a direct result of stretch or ECM reorganization by fibroblasts in the cultures.

Phasic stretching has also been used to stimulate cells in culture. Phasic stretching of a ring‐shaped tissue‐engineered construct seeded with C2C12 cells resulted in more regularly aligned muscle fibers compared to the nonstretched condition.^[^ ^372^ ^]^ Phasic stretch at 1.5–2 Hz of collagen gels containing cardiomyocytes led to increased cellular alignment, RNA and protein synthesis, cell size, expression of cardiac marker genes, and longer myofilaments.^[^ ^373^ ^]^ However, another study found that a variety of stretch stimuli decreased the maturation of C2C12 cells,^[^ ^374^ ^]^ suggesting that the effects of stretch stimuli may be highly dependent on the specific stretch protocol used and/or other features of the culture conditions. As an alternative to direct mechanical stimulation, certain electroresponsive polymers can translate electrical currents into mechanical stimuli that improve smooth muscle cell distribution throughout the scaffold, cell alignment,^[^ ^375^ ^]^ collagen deposition, compaction of the tissue construct, and tensile strength.^[^ ^376^ ^]^ The interplay between mechanical stimuli, cell type, and other features of the culture conditions—and the impacts of these variables on cell behavior and maturation—remains to be fully understood. In the context of CM, application of stretch stimuli to long‐term cultures remains underexplored as a means of inducing both construct growth and fiber alignment in complex whole‐cut products.

#### 6.2.6 Electrical Stimulation and Muscle‐Neuron Cocultures

Electrical stimulation substantially improved the maturation of bovine myotubes cultured in aligned scaffolds composed of either collagen or a mixture of fibrin and Matrigel.^[^ ^119^ ^]^ Electrical stimulation also accelerated the maturation process in 2D and 3D constructs seeded with C2C12 myoblasts or primary muscle progenitors, with a greater effect in the primary cells.^[^ ^226^ ^]^ Another study found a similar effect in primary mouse myoblasts, although this effect depended on the substrate coating and stiffness and was fairly mild, leading the authors to suggest that electrical stimulation might be dispensable for engineering mature muscle tissue.^[^ ^270^ ^]^ A combination of media perfusion and electrical stimulation led to better tissue organization and cardiac marker gene expression in rat primary cardiac cells under conditions where the tissue constructs were allowed to freely contract in response to the electrical stimulation.^[^ ^377^ ^]^

In the context of CM, energy input is likely to be a key factor in determining both the cost of production^[^ ^22^ ^]^ and the carbon footprint^[^ ^19^ ^]^ of the final product. Therefore, there may be an advantage to using passive stretch stimuli such as those generated in a clamped gel compared to either phasic stretch stimuli or electrical stimulation.

A recent article investigating cocultures of myocytes and motor neurons on aligned nanofiber scaffolds found that the presence of the motor neurons promoted increased myocyte fusion compared to myocytes cultured alone.^[^ ^33^ ^]^ Another recent paper from Atala and groups cocultured human muscle progenitor cells with human neural stem cells in a ratio optimized for myotube formation and 3D bioprinted these cells together in a hydrogel formed of fibrinogen, gelatin, and hyaluronic acid. Similar to the previous study, incorporating the neural cells improved muscle differentiation and maturation.^[^ ^34^ ^]^ Conditioned media from neuronal cultures were found to contain a variety of growth factors with known effects on muscle cell proliferation and maturation.^[^ ^34^ ^]^ Other groups have similarly demonstrated increased muscle hypertrophy in the presence of motor neurons.^[^ ^378^ ^]^ The effects described in these studies are thought to result from a combination of neurotransmitter release leading to muscle fiber contraction and resulting effects similar to those seen with electrical stimulation, as well as effects of various other factors secreted from the neurons.

### 6.3 Perfusable Vascular‐Mimicking Networks

In the body, only tissues with low cell density and metabolism, such as the cornea and cartilage, can survive without vascularization.^[^ ^379^ ^]^ For CM, the main reason for including a vascular or vascular‐like network is to provide oxygen and nutrients to—and remove wastes from—all of the cells within a large piece of tissue. Techniques such as 3D printing,^[^ ^380^ ^]^ micromolding, and embedding rapidly dissolving structures within cell‐laden hydrogels all provide promising approaches to engineering perfusable structures.^[^ ^379^ ^]^ Additional approaches to in vitro vascularization have been reviewed elsewhere.^[^ ^23^, ^30^, ^381^ ^]^ As discussed in Section 5.4, decellularized plants are another potential solution to the problem of oxygen and nutrient transport, as they are equipped with a built‐in vasculature that mirrors the branching patterns found in animal tissues. Methods for producing vascular‐like networks are discussed below, along with the question of whether networks with a substantially lower density than those found in vivo might suffice for cultivated meat and the use of perfusion bioreactors for scale‐up of 3D tissues.

#### 6.3.1 Sacrificial Materials

One potential strategy for creating vasculature‐mimicking channels is to form the channels from a sacrificial material that can be washed away once the surrounding scaffold and the cells are in place. One research team at MIT developed a versatile perfusable scaffold system by 3D printing a dissolvable lattice of perfusable channels using a brittle carbohydrate‐based glass (a mixture of dextran, glucose, and sucrose), casting a cell‐laden hydrogel surrounding the lattice, and then dissolving the glass lattice with warm culture medium, leaving behind perfusable channels.^[^ ^228^ ^]^ In this study, primary hepatocytes embedded at a density of 24 × 10^6^ cells mL^−1^ were able to survive to some extent in these scaffolds up to a distance of ≈0.5 mm from the nearest channel, with the highest density of live cells within ≈0.1–0.2 mm. Similarly, using the sacrificial writing into functional tissue technique, vasculature was created by embedded 3D printing using a sacrificial (fugitive) ink that is removed following curing of the overall structure, using a high density of cells via multicellular spheroids or organoids as the “organ building blocks.”^[^ ^242^ ^]^ They formed perfusable cardiac tissue for eight days, which matured and was able to beat. As an alternative to traditional 3D printing, variations on the concept of additive manufacturing—including 2‐photon polymerization^[^ ^264^ ^]^ and laser sintering^[^ ^382^ ^]^—may also be used to create sacrificial vascular structures to be embedded into a scaffold and then dissolved. Importantly, in muscle constructs containing channels formed from bioprinting sacrificial gelatin, muscle fibers were able to expand over time and take over some of the spaces previously occupied by the channels, resulting in dense tissue.^[^ ^241^ ^]^ This observation suggests two things for the production of CM: first, that low‐density bioprinted tissue with large channels can mature into higher‐density tissues under the right conditions, and second, that there may be some risk of channels intended to facilitate oxygen and nutrient transport closing up over the course of the maturation process. Consistent with this, engineered cartilage constructs grown on fibrous PGA became dramatically less permeable over several weeks of culture and displayed a tight correlation between the total amount of cells, glycosaminoglycans, and collagen found in the tissue and the permeability to glucose and dextran.^[^ ^383^ ^]^ The right balance of vascularization will need to be found to generate tissues dense enough to recapitulate the organoleptic properties of meat while avoiding the formation of necrotic cores.

#### 6.3.2 Nonsacrificial Methods

Several methods that avoid the use of sacrificial materials have also been described. The “tissue in a tube” strategy relies on the contraction of a collagen‐based bioink within a tube, leaving a hollow, media‐filled space surrounding the construct, which may be used for perfusion.^[^ ^304^ ^]^ This method has been tested successfully with endothelial, epithelial, muscle, osteoblast, and neuronal cells and is suitable for the production of tubular constructs several centimeters in length, although the diameter is limited by the fact that channels are not present in the construct's interior. Molding of scaffolds around linear wire arrays has been used as a strategy for introducing vascular‐like channels, which were demonstrated to improve both fibroblast seeding and oxygen and nutrient delivery within small‐scale constructs.^[^ ^161^ ^]^ Similar array‐based strategies have been used for tendon and spinal cord tissue engineering.^[^ ^384^, ^385^ ^]^ Using a stereolithography‐based approach in which a photoabsorber compound was added to the prehydrogel solution to block excess light penetration, Grigoryan et al. managed to directly print hydrogel constructs containing perfusable channels without the use of any sacrificial material.^[^ ^386^ ^]^ Interestingly, the photoabsorbers tested included tartrazine, curcumin, and anthocyanin, which are already approved for and commonly used in food applications.^[^ ^386^ ^]^ Perfusable vascular‐like channels were also demonstrated using the ExCeL technique, in which sheets of paper are pretreated with cross‐linker solution and cut to create the desired structures, after which cells are printed onto the paper and then multiple sheets are stacked to create 3D constructs.^[^ ^245^ ^]^

#### 6.3.3 Endothelial Cells

While endothelial cells themselves are likely not directly required for oxygen and nutrient transport within CM as long as a suitable set of channels are provided, it may be possible to reduce the need for fine patterning of vascular‐like structures by taking advantage of these cells’ tendencies toward forming tubes and branched structures. One suggested scheme for bioprinting tissue‐engineered organs involves the use of spheroids containing endothelial and smooth muscle cells, as well as other cell types relevant to the organ in question, to form a complete vascular tree with multiple vessel diameters.^[^ ^181^ ^]^ These spheroids, once printed, are expected to self‐organize into a functional vascular network. This self‐organizing principle appears to have some experimental support; hydrogels containing a mixture of endothelial cells and satellite cell‐derived myoblasts were able to form aligned fibers 1.5 mm thick containing capillary‐like endothelial networks after one week of culture.^[^ ^387^ ^]^ An alternate version of this method in which the myoblasts were first cultured on their own for some time, followed by the addition of an outer layer of endothelial cells, resulted in vascular networks that were longer, more branched, and better connected.^[^ ^160^ ^]^ Self‐organization of endothelial cells into tubules appears to be enhanced by co‐culture with MSCs, and the strength of this enhancement is dependent on the scaffold material.^[^ ^388^ ^]^ 3D printing of cardiac muscle cells and endothelial cells has been demonstrated for miniature human hearts as a proof of concept.^[^ ^389^ ^]^

#### 6.3.4 Required Density of Vascular‐Like Channels

A key question for CM is to what extent it will be necessary to faithfully mimic the structure and function of the vasculature found in living animals. A review paper from Vladimir Mironov's group describes vascularization of thick tissues as a key unsolved challenge in tissue engineering.^[^ ^181^ ^]^ A key point from this review that may be relevant to CM is the importance of vascular channels with a range of sizes. Larger‐diameter vessels are necessary to carry blood (or in the case of CM, culture media) across the full thickness of the construct, and smaller capillary‐like channels are necessary to carry blood or media close enough to each cell that oxygen and nutrient diffusion can reach all cells within the scaffold. Histological characterization of human vastus lateralis muscle showed the majority of muscle fibers in direct and close contact (<10 µm) with at least one capillary, and often several, and an overall capillary‐to‐fiber ratio between 1 and 2.^[^ ^390^ ^]^ The same study revealed that the cross‐sectional area of the average fiber ranged from ≈800 to 1600 µm^2^, which translates to a diameter of 32–45 µm, assuming roughly circular fibers. This is consistent with estimates of typical muscle fiber diameters of 10–50 µm^[^ ^35^ ^]^ or 10–100 µm.^[^ ^29^ ^]^ The upper limit for mass transfer of oxygen has been estimated as ≈200 µm,^[^ ^391^ ^]^ or about 2–20 muscle fiber diameters. This difference is not terribly surprising, given that muscles in vivo must be capable of supporting not only the oxygen requirements of cells at rest but also those during high‐intensity exercise, which may be up to 100‐fold higher.^[^ ^392^ ^]^ Because CM does not need to support the needs of a living animal, its vascularization requirements may be considerably lower than those of naturally occurring muscle tissue, and therefore a less complex vasculature may suffice. In tissue‐engineered C2C12 muscle constructs growth in a hollow fiber bioreactor (HFB), cells remained viable at distances up to 98 µm from the nearest fiber.^[^ ^393^ ^]^ At the very least, CM scaffolds will need to provide pores or channels for oxygen and nutrient transport no more than about 100–200 µm away from any given point in the tissue construct. The required density of channels or pores may also depend on whether specific oxygen carriers, such as recombinant hemoglobin, are used in the culture media.^[^ ^394^, ^395^, ^396^ ^]^

#### 6.3.5 Perfusion of Scaffolded Constructs in Hollow Fiber Bioreactors

For successful commercial production of CM, it will be necessary to select not only an appropriate scaffold but also an appropriate bioreactor. The choice of bioreactor may depend in part on the choice of scaffold and vice versa. General considerations for the use of bioreactors in tissue engineering have been reviewed elsewhere.^[^ ^166^, ^391^ ^]^ Because the ability to efficiently perfuse media through a cell‐laden scaffold will become increasingly important as tissue engineering techniques are scaled up for use with CM, innovations in perfusion bioreactors will be a necessary complement to innovations in scaffolding. Although small scaffold constructs can be simply grown in suspension, collisions between scaffolds or with the bioreactor itself have been shown to limit cell density and growth rate.^[^ ^397^, ^398^ ^]^ In addition, the necessity of medium perfusion through the scaffold will increase with the size of the construct, as passive diffusion through pores or channels may be insufficient for oxygen and nutrient transport. For example, hydrogels with vascular‐mimicking channels 750 µm in diameter were able to support the survival of hepatocytes, but only when the construct was actively perfused.^[^ ^228^ ^]^ Furthermore, perfusion may influence differentiation via shear stress.^[^ ^399^ ^]^

Questions related to perfusion, scaffold permeability, fluid flows, nutrient mass transfer, and stressors within scaffolds may be investigated with the aid of computational modeling. Computational models can decrease the time and cost burden of running physical experiments and inform the optimized design of scaffolds, perfusion bioreactors, or sensory equipment. Computational approaches previously applied to bioreactor optimization for bone tissue engineering^[^ ^400^ ^]^ can be adopted by CM investigators.^[^ ^401^ ^]^ However, the high complexity of multiple cell types growing within nonuniform scaffolds to create a diverse set of CM products will be computationally intensive and pose new challenges that may require multiscale approaches that have lower computational costs.^[^ ^402^ ^]^ The Cultivated Meat Modeling Consortium^[^ ^403^ ^]^ provides a forum for computational modelers to understand and address the most pressing scaffolding and perfusion‐based challenges in collaboration with CM manufacturers.

A recent review of bioreactors specifically focused on their applications in CM noted that HFBs, in which culture medium is actively perfused through a dense array of permeable fibers, are by far the most capable of supporting high‐density culture and therefore require much smaller volumes to produce a given amount of cell mass.^[^ ^83^ ^]^ Cells are grown on the outside of these fibers, thus ensuring a reliable supply of oxygen and nutrients, as well as the removal of waste products. These systems are similar to what is used for kidney dialysis machines^[^ ^404^, ^405^ ^]^ as well as large‐scale water treatment,^[^ ^406^, ^407^ ^]^ pointing to feasibility for scaling up the technology,^[^ ^408^ ^]^ and they have been demonstrated for mammalian cell culture in a laboratory‐scale proof‐of‐concept system.^[^ ^409^ ^]^ The use of HFBs was also modeled in a life cycle assessment of CM.^[^ ^410^ ^]^ Other perfusion systems, such as packed bed and fluidized bed bioreactors, are more readily scalable^[^ ^330^ ^]^ and may be useful for the proliferation phase of CM production,^[^ ^83^ ^]^ but operate by perfusing media around particles (i.e., cells, cell aggregates, or microcarriers) rather than through a scaffold. Thus, this review limits its focus to HFBs as they are well‐suited to the task of perfusing media through a dense, tissue‐like construct.

Cellular Agriculture Ltd. is developing HFBs that slowly perfuse culture medium through parallel tubes (fibers) of a porous polymeric material, delivering a carefully controlled amount of oxygen and nutrients to the surrounding cells. FutureMeat Technologies, another academic spin‐out, has published a patent on perfusion bioreactor systems for growing CM.^[^ ^141^ ^]^ The patent describes growing cells or tissues in a chamber through which serum‐free medium is perfused and subsequently recycled.

An HFB featuring cellulose triacetate fibers was able to maintain C2C12 cells over a period of 7 days and support differentiation into multinucleated myotubes expressing MHC and tropomyosin.^[^ ^393^ ^]^ Although the resulting tissues exhibited high cell density within the cell culture space, the fibers themselves represented a majority of the space within the total construct, reducing the overall cell density of the construct considerably. Additional studies have also reported small‐scale growth of C2C12 myoblasts in HFBs, either as single cells^[^ ^411^ ^]^ or as spheroids.^[^ ^412^ ^]^

Despite the advantages in achievable cell density offered by HFBs, they also suffer from a tendency to undergo membrane fouling—largely due to the deposition of albumin and other proteins on the fiber surface—as well as compaction of the fiber material, leading to decreases in oxygen and nutrient flux over time.^[^ ^413^ ^]^ Studies examining the use of these bioreactor designs for CM applications should carefully assess membrane materials, pore sizes, and operating conditions to minimize the effects of fouling.^[^ ^413^ ^]^

HFBs may be further improved for use as meat cultivators through optimization of the design itself and the operating parameters. For example, alternating the flow direction led to a threefold increase in the number of cells produced within an HFB as well as a more even cell distribution within the reactor, as compared to unidirectional flow.^[^ ^414^ ^]^ Variations on the traditional hollow fiber design are also possible: for example, a system with two sets of fibers for media perfusion in opposite directions designed to minimize gradients within the tissue and a third set dedicated to gas supply was investigated for adipose tissue engineering with promising results.^[^ ^415^ ^]^ Fabrication of hollow fibers from biodegradable materials such as PLA^[^ ^416^, ^417^ ^]^ or PCL^[^ ^418^ ^]^ has been demonstrated at a small scale. Rolled sheets of channel‐bearing PCL in combination with a fibrin hydrogel have been reported to be able to support the proliferation of C2C12 myoblasts within a perfusion bioreactor.^[^ ^195^ ^]^ Optimization of such fibers for use in larger formats and with an optimized degradation rate might improve the ability to produce whole‐cut CM without the need for complicated harvest procedures, effectively turning the hollow fibers themselves into part of the scaffold.

At scale, perfusion bioreactors will likely contribute substantially to the overall cost of production of CM,^[^ ^22^, ^419^ ^]^ with sterility maintenance acting as a major cost driver.^[^ ^419^ ^]^ The ability of perfusion bioreactors, and especially HFBs, to support high‐density cell culture^[^ ^83^ ^]^ could in theory lead to cost savings and environmental benefits. However, unlike other designs such as stirred‐tank^[^ ^420^ ^]^ or airlift bioreactors,^[^ ^421^ ^]^ large‐scale bioreactors capable of perfusing media through a channeled or porous construct remain theoretical. Significant innovations in perfusion bioreactor design will be necessary for these technologies to be adopted by the high‐volume CM industry.^[^ ^419^ ^]^

### 6.4 Growing Large and Heterogenous “Whole‐Cut” Tissues

Several recent studies have reported successful attempts at engineering muscle at the scale of several mm to several cm, including CM prototypes which in some respects resemble whole‐cut tissues (**Table** **7**). Histological, instrumental textural, and sensory analyses have revealed a reasonable degree of similarity to conventional meat. Macroscopic engineered muscle tissues that could reasonably be called “meat”—though thus far, very small pieces of meat only—can be cultivated today within academic settings, and larger pieces of CM have been demonstrated by for‐profit companies.^[^ ^17^ ^]^

**Table 7:** Summary of studies that have produced CM prototypes on the mm‐ or cm‐scale

Cells	Scaffold	Dimensions	Comparisons to conventional meat	Reference
Rabbit muscle and bovine smooth muscle	Electrospun gelatin	≈4 cm^2^ area, 1.5 mm thick	Texture profile analysis more closely resembled ground beef than whole muscle from rabbit or beef. Muscle fibers were well‐aligned, but constructs lacked the densely packed tissue architecture of conventional whole‐cut meat products.	^[^ ^59^ ^]^
Bovine satellite cells	Fibrin hydrogel	13 × 1.4 × 2.3 mm, 39 mg weight	L*a*b color values of constructs cultured with hemoglobin or myoglobin were similar to those of cooked but not raw beef.	^[^ ^115^ ^]^
Bovine satellite, endothelial, and smooth muscle cells	Textured soy protein	6 mm diameter, 1–2 mm thick	Textural properties (Young's modulus and ultimate tensile strength) were somewhat similar to bovine muscle. Described by taste testers as having “a pleasant meaty flavor and sensorial attributes, achieving a typical meat bite and texture.”	^[^ ^123^ ^]^
Bovine muscle, adipose, endothelial cells	Tendon‐gel integrated printing: fibrinogen and Matrigel‐based bioink with gelatin “tendons”	5 × 5 × 10 mm	Visual comparison only. Distribution of printed fibers composed of different cell types was modeled off of histological measurements of the cell type distribution in conventional Wagyu beef.	^[^ ^121^ ^]^
Bovine myocytes	Stacked fibrin and Matrigel hydrogels	8 × 10 × 7 mm	Breaking force similar to that of conventional beef.	^[^ ^119^ ^]^
C2C12 myoblasts, 3T3‐L1 preadipocytes	Scaffold‐free cell sheets	1 cm diameter, 18 cell sheets (≈1–2 mm) thick, or 2.5 cm diameter, six sheets thick	No direct comparison to conventional meat.	^[^ ^120^ ^]^

Many of the earliest CM products on the market will likely be burgers, sausages, or other unstructured products. However, for CM to have a major impact on the food system, it is necessary to also develop methods for reproducing the complex, heterogeneous structures that give foods like steaks and fish fillets their characteristic organoleptic properties. For most whole‐cut products, muscle fiber alignment will play an important role in defining the sensory characteristics of the meat as well as serving as a differentiation and maturation cue (see Section 6.2). Creating larger pieces of meat will require innovations that facilitate nutrient and oxygen transport in a simple, cost‐effective, and scalable manner (see Section 6.3). At the very least, CM products will need to include appropriate ratios of muscle and fat, and in the case of whole cuts will need to feature these different cell types in the correct 3D layout. Other tissues such as bone, tendon, and cartilage make an important contribution to the experience of eating certain cuts of meat. Although such cuts are likely not the “low hanging fruit” that the CM industry will target for early products, it is worth considering some of the unique challenges they may pose. A recent review^[^ ^422^ ^]^ suggested that combinations of two or more biomanufacturing techniques may be the most promising category of approaches to the challenge of engineering large, complex, and hierarchical constructs with the correct organization on multiple scales. The following sections will discuss the challenge of producing complex 3D tissues containing both muscle and fat cells—as well as potentially other cell types relevant to certain cuts of meat—and the use of stiffness cues as a means of steering cells down particular differentiation pathways in a spatially defined manner.

#### 6.4.1 Combining Muscle and Fat

Fat is an important contributor to both the nutritional value and the organoleptic properties of meat.^[^ ^29^ ^]^ As with muscle, engineering of fat tissues has been investigated for biomedical applications.^[^ ^222^, ^227^, ^272^, ^327^, ^415^, ^423^, ^424^ ^]^ For unstructured CM products, growing fat and muscle separately and then combining them in a final processing step may be the simplest and most cost‐effective solution. In the case of whole‐cut products, muscle fibers, adipocytes, and connective tissue will need to be formed into a cohesive tissue under conditions that are compatible with viability and maturation of all cell types.

Fat and muscle cells both secrete various factors that can impact one another's proliferation, differentiation, and metabolism.^[^ ^425^ ^]^ This relationship is complex. For example, it has been reported that co‐culture with fat cells both promotes^[^ ^426^ ^]^ and suppresses^[^ ^427^ ^]^ differentiation of muscle cells. The source of the cells may matter as well. In one study, coculture with adipocytes derived from visceral adipose tissue decreased myotube thickness and expression of muscle‐specific genes, whereas those derived from subcutaneous adipose tissue had less pronounced effects.^[^ ^428^ ^]^

Furthermore, fat and muscle in conventional meat are distributed heterogeneously, and the cell type distribution is important for the quality of the final product. For example, in highly marbled meat such as Wagyu beef, the presence of intramuscular fat deposits serves to disorganize the connective tissue, thereby leading to a more tender product.^[^ ^46^ ^]^ The creation of realistic whole‐cut CM will require methods for producing a heterogeneous and finely tuned distribution of fat. Several studies have reported the successful 2D coculture of myogenic and adipogenic cells from agriculturally relevant species,^[^ ^429^, ^430^, ^431^, ^432^ ^]^ while others have reported small‐scale 3D cultures using human or mouse cells.^[^ ^120^, ^428^, ^433^ ^]^ In addition, the company Ants Innovate is developing a scaffolding system to support the growth of marbled meat containing muscle cells and fat. They have filed a patent on their biomaterial system,^[^ ^434^ ^]^ and the founders have recently published an article using a cellulose sponge for organoid culture.^[^ ^435^ ^]^ A recent review discusses the main considerations relevant to cultivated fat, both as an ingredient and as a component of CM, including potential approaches to scaffolding for adipocyte growth and maturation.^[^ ^37^ ^]^

#### 6.4.2 Incorporating Bone, Tendon, and Cartilage

For certain cuts that incorporate bone, tendon, or cartilage, additional cell types will need to be incorporated or realistically mimicked, again in the correct 3D distribution. Scaffolds derived from a combination of collagen and hydroxyapatite have been investigated for use as bone grafts and can support the adhesion and proliferation of fibroblasts.^[^ ^436^ ^]^ Growth of bone tissue or osteogenic cells on decellularized apple^[^ ^326^ ^]^ and carrot^[^ ^327^ ^]^ scaffolds, PLGA hollow fiber membranes,^[^ ^196^ ^]^ soda bread,^[^ ^191^ ^]^ RADA16 hydrogels,^[^ ^237^ ^]^ collagen hydrogels,^[^ ^304^ ^]^ and laser sintered PCL^[^ ^281^ ^]^ has also been demonstrated. Engineering of tendon^[^ ^327^, ^384^, ^437^ ^]^ and cartilage^[^ ^438^, ^439^ ^]^ tissue are also active areas of investigation for biomedical applications. While a detailed discussion of the engineering of bone, tendon, and cartilage tissue is outside the scope of this review, it is interesting to speculate how these methods might be adapted for the creation of more complex cuts of CM. An obvious prerequisite for this to occur at commercial scale will be a dramatic reduction in the cost of producing CM. Achieving price parity with conventional meat is already a challenge,^[^ ^22^, ^419^ ^]^ and the cost of engineering an additional type of tissue will need to be commercially justified. This may be especially difficult in the case of bone‐in products such as chicken wings and ribs, in which the bones ultimately will not be consumed. Therefore, it is likely that the first generation of CM products will likely consist of cuts that require only muscle, fat, and connective tissue. Bone‐in cuts of CM may be developed in the relatively near future in which the bones are made from cheaper alternative materials since cellular fidelity is not critical for these components of the product.

#### 6.4.3 Stiffness Cues

ECM stiffness has been identified as an important cue for cellular differentiation^[^ ^271^, ^440^, ^441^ ^]^ and thus represents an opportunity for spatially fine‐tuning the concentrations of different cell types in a heterogeneous manner. When MSC‐laden alginate bioinks of varying stiffnesses were printed together in a nonhomogenous pattern, the softer regions of the construct tended to differentiate toward the adipogenic lineage, whereas cells in the stiffer regions were more likely to choose the osteogenic lineage.^[^ ^222^ ^]^ By further tuning the stiffness of the bioinks, their 3D distribution, and the culture conditions, a similar strategy could be used to produce whole cuts of CM including muscle and fat cells, as well as potentially other cell types if desired. Previously reported measurements of the mechanical properties of tissues or meat (Table 2) might inform such efforts.

## 7 Considerations Specific to Food Products

CM is first and foremost a food product. As such, considerations related to the organoleptic properties of the final product, whether and where it can be sold given existing regulations, acceptance by consumers, and food safety must be top of mind when making decisions about the production process. These issues are discussed in further detail below.

### 7.1 Engineering Taste and Nutrition

The relationship between meat intake and health is complex, difficult to interpret, and often confounded by other factors.^[^ ^7^ ^]^ This introduces both challenges and opportunities for producers of CM. For these products to be welcomed as replacements for conventional products, the health benefits of meat, including amino acid profiles and micronutrients, will need to be faithfully recapitulated. Both nutritional and organoleptic properties are likely to depend on the extent of differentiation and maturation; protein content in C2C12 cells increased by ≈50% when differentiation was induced.^[^ ^442^ ^]^ Similarly, Young's modulus of cultured myoblasts has been shown to increase substantially when the cells are induced to differentiate, from 11.5 kPa for myoblasts to 45.3 kPa for myofibers after eight days of differentiation,^[^ ^443^ ^]^ which has important implications for the texture of CM.

At the same time, CM presents an opportunity to not only match but improve upon the properties of conventional meat while mitigating some of the negative health impacts of meat consumption,^[^ ^94^ ^]^ such as the link between high levels of processed meat consumption and colorectal cancer.^[^ ^7^ ^]^ Recent years have seen an increase in the number of companies focused on the use of bioengineering in food with better health outcomes as an explicit goal, with largely positive reception from the public.^[^ ^444^ ^]^ For example, a consortium led by BioTech Foods recently received funding from the Spanish government for a project aimed at producing CM products with less saturated fat and more “healthy” fats, which the researchers predict will reduce the risk of colon cancer and dyslipidemia.^[^ ^445^ ^]^ Genetic engineering strategies aimed at increasing carotenoid content in cultivated red meat have already been successfully demonstrated on a small scale.^[^ ^110^ ^]^

### 7.2 Regulation

The first commercial product launches will coincide with CM manufacturers entering pilot‐scale facilities, as evidenced by the approval and subsequent commercialization of the first CM product in Singapore in December of 2020.^[^ ^17^ ^]^ Many other regulatory bodies are also working on developing safety and regulatory frameworks for CM. At this moment, Singapore, Canada, Australia/New Zealand, the United Kingdom, and the European Union all currently have an applicable regulatory framework relevant to CM; the United States, Japan, and Israel have an expressed interest in CM with regulatory updates pending; and India, Brazil, and China are monitoring global progress with an eye to creating a path to market.^[^ ^20^ ^]^ In general, regulatory guidances are anticipated to lie at the nexus of those established for the food and biomedical industries. Details on the regulatory structures for some of these regions have been covered elsewhere.^[^ ^88^, ^446^ ^]^

Because scaffolds are anticipated to be made from food‐safe, edible, or biodegradable materials, their inclusion in products is not expected to pose significant regulatory barriers. In some instances, CM manufacturers may wish to use genetically modified (GM) scaffolding materials or incorporate synthetic or engineered polymers and peptides. Any genetically modified or engineered material, polymer, or peptide would likely be subject to existing GM regulations and labeling depending on jurisdiction. Manufacturers that use synthetic materials may also be subject to additional regulatory oversight, including the demonstration of safety for novel synthetic materials that have not yet been introduced in the food system. Meeting additional regulatory requirements would translate into longer review periods and higher compliance costs, and could negatively influence consumer perception of CM products. Together, this may provide an incentive for CM manufacturers to use non‐GM scaffolding materials that are covered under current food safety regulations.

### 7.3 Assessing Quality and Safety

Different products are likely to contain variable percentages of scaffold materials as a fraction of final product weight. In some instances, a scaffold may biodegrade to undetectable levels in the final product. Scaffolds that remain integrated into the final product will likely be subject to food safety regulations as an ingredient or additive, depending on the level present and local regulatory definitions. Scaffold and biomaterial suppliers should therefore manufacture scaffolds according to general HACCP principles and under food GMP conditions to ensure that controls are in place to prevent unintentional food safety hazards such as allergen cross‐contamination.^[^ ^447^ ^]^

Depending on the method of construction, a scaffold could be contaminated by non‐food‐safe chemical solvents or may include other non‐food‐safe components such as some photoinitiators and chemical cross‐linking agents used for scaffold polymerization.^[^ ^448^ ^]^ Further research by scaffold manufacturers may be needed to avoid the use of potentially hazardous solvents and to develop food‐safe polymerization agents. If a scaffold is purposed to degrade or transform throughout the manufacturing process, potential degradation by‐products may require new safety assessments. Physicochemical transformations such as oxidation, degradation, or enzymatic processing of synthetic polymers could also affect product quality and give rise to new safety risks.^[^ ^449^ ^]^ CM manufacturers should therefore consider the safety implications for all scaffolding inputs and materials processing agents prior to their use in order to mitigate downstream food safety risks.

## 8 Conclusions and Outlook

A variety of approaches have been proposed or explored for recapitulating the structure of muscle tissue. These include a diverse array of scaffold biomaterials and methods, as well as approaches such as organoids or cell sheets that avoid the use of scaffolds entirely. There will likely not be a one‐size‐fits‐all approach to scaffolding for CM but rather a variety of solutions for a variety of different end products.

Protein‐ or peptide‐based scaffolds, including ECM proteins and self‐assembling peptides, have a host of advantages, but there is a lack of low‐cost and scaled sources appropriate for use in CM. Production using plants, bacteria, yeast, animal cell culture, or cell‐free systems may allow for the cost‐effective use of both ECM protein‐based scaffolds and self‐assembling peptides. In parallel, it will be important to identify the most critical ECM molecules for use in scaffolding, with attention to differences between species and cell types. Costs may be further reduced by exploring combinations of materials that include a small percentage of ECM proteins or self‐assembling peptides together with lower‐cost materials to form the bulk of the scaffold structure.^[^ ^122^, ^126^, ^240^, ^255^ ^]^

Synthetic materials including PLA, PLGA, PEG, and PCL are currently unsuitable for use as CM scaffolds intended to remain in the final product, primarily due to the need for scaffold materials to either biodegrade rapidly (i.e., complete degradation during the culture period or postharvest aging) into harmless byproducts or be suitable for direct consumption. Unless new innovations can address these challenges, these materials will be appropriate only as microcarriers or other temporary scaffolds. Although reported degradation rates for these materials are unacceptably long, the degradation rate of PCL varies substantially depending on factors including initial molecular weight, construct geometry, pH, and temperature.^[^ ^290^ ^]^ Degradation could perhaps be accelerated sufficiently for use in CM, e.g., by using low molecular weight forms of these polymers as very thin fibers. In parallel, the identification of novel polymers with faster degradation and other desirable characteristics could yield better results. Reactions during cooking, including Maillard reactions, lipid oxidation, and vitamin decomposition, produce a variety of meat‐associated flavor compounds.^[^ ^450^ ^]^ Thus, combining some of these precursors into edible synthetic polymer scaffolds could create functional scaffolds that release these compounds upon their eventual breakdown and thereby improve the flavor of the final meat product. For any polymer‐based scaffold, it will be necessary to characterize the degradation profile, the tendency of the resulting monomers to be washed out of or retained in the final product, and the food safety and organoleptic implications of any remaining scaffold material or monomers. In addition, the food safety implications of any solvents or polymerization reagents used will need to be carefully considered.

Plant‐ and fungus‐derived materials are a promising category of potential scaffolding materials but present some challenges that further research must address. Methods such as particle leaching, melt molding, freeze drying, and gas foaming that have been extensively investigated with nonedible scaffolding materials should be further investigated in the context of plant‐ and fungus‐derived edible biomaterials.^[^ ^122^, ^274^, ^334^ ^]^ The use of decellularized plant tissues^[^ ^81^, ^118^, ^131^ ^]^ is a promising area due to their inherently vascularized structure. However, additional innovations are needed concerning the development of scalable, sustainable, and food‐safe decellularization techniques, as well as methods for combining vascularized tissues produced using such scaffolds into larger whole‐cut CM products.

Methods to improve cell adhesion to a variety of materials have the potential to improve these materials’ applicability as CM scaffolds. Materials such as alginate, PCL, and PLGA could be enhanced through functionalization with ECM motifs or other methods, especially those compatible with existing food safety standards such as approved food ingredients that happen to contain the relevant adhesion motifs. The use of ECM motifs in combination with CBDs^[^ ^344^ ^]^ should be further explored to functionalize plant‐based scaffolds. As an alternative to direct functionalization, the ability of recombinant ECM proteins—particularly vitronectin and fibronectin^[^ ^249^ ^]^—added to serum‐free culture medium to improve adhesion to a variety of CM‐compatible scaffold materials should be assessed. For all these methods, the feasibility of reducing costs as production is scaled up should be further assessed as more knowledge is gained, and these costs weighed against the efficacy of each method.

It will also be necessary to pursue innovations related to bioreactor and bioprocess design, including the optimization of membrane materials, pore sizes, and operating conditions to minimize the effects of fouling in HFBs designed for CM.^[^ ^413^ ^]^ Researchers should also investigate how bioreactors could be designed to simultaneously perfuse media through complex tissues and deliver stretch or other stimuli relevant to muscle maturation to enhance alignment and growth in long‐term cultures of whole‐cut CM. Furthermore, the use of 2D cell sheets is an interesting concept with the advantage that no exogenous scaffold material is introduced into the construct.^[^ ^120^, ^188^ ^]^ However, developing novel bioreactors and automated assembly methods will be necessary for this technique to apply to scaled CM production.^[^ ^120^ ^]^ For all of these approaches, it will be critical for bioreactor designs to simultaneously take into account cost considerations and the necessity of reliably maintaining sterile operating conditions.^[^ ^419^ ^]^

Methods for producing muscle fiber alignment and spatial heterogeneity, including acoustic cell patterning, bioprinting, lithography, embossing, micromolding, and incorporation of growth factors into scaffolds in defined patterns, require further exploration. Novel combinations of techniques should be seriously explored, as this may make it possible to combine the strengths of multiple biofabrication or structuring techniques,^[^ ^422^ ^]^ thereby ultimately producing a better product. To facilitate comparisons across studies, we recommend using metrics for muscle tissue maturation that can be easily and quantitatively compared, such as fusion indices based on the percentage of myotubes with a threshold number of nuclei (or better yet, the distribution of myotubes with various numbers of nuclei), myotube width, and myotube length.^[^ ^197^, ^208^, ^274^ ^]^ Such a strategy may reduce duplication of effort and allow the field to more quickly align on the most effective methods for inducing differentiation and maturation. It will be important to thoroughly investigate processes such as tissue compaction and resulting reductions in the widths of pores or perfusable channels in the maturing tissue,^[^ ^241^ ^]^ as well as ECM deposition by fibroblasts and other cells. Understanding the factors that influence the rate and extent of both processes will make it possible to optimize for high‐density tissues while avoiding necrotic cores and to tune textural properties by ensuring that the amount, composition, and organization of connective tissue in the final product is correct. It may be desirable to control the rate of ECM remodeling by manipulating the activity of MMPs and TIMPs^[^ ^43^ ^]^ if it proves to be the case that early whole‐cut products suffer from too much, too little, or poorly organized connective tissue.

Meeting nutritional expectations will require considering beneficial compounds that are not produced by animal cells but rather consumed through animals’ diets. For example, fish are well known as excellent sources of polyunsaturated omega‐3 fatty acids, but marine fish primarily accumulate these from food sources such as algae. Freshwater fish have less access to the long‐chain omega‐3s DHA and EPA in their diet and can produce them in small quantities from the precursor ALA within their livers.^[^ ^451^ ^]^ Scholefield and Schuller found that cultured bluefin tuna cells accumulated fatty acids present in the culture medium, but there was no evidence that the cells synthesized DHA or EPA from precursors ALA and LNA.^[^ ^452^ ^]^ Therefore, supplementation of DHA or EPA through the culture media or the scaffold could increase omega‐3 content in cultivated seafood. Alternatively, coculture with other cell types or genetic engineering could help recapitulate conventional fatty acid profiles. Similar strategies will apply for other beneficial compounds acquired through the animal's diet or produced by gut microbes, such as vitamin B12.^[^ ^453^ ^]^

Producing CM with the desired organoleptic properties will depend on achieving the right balance of sufficiently mature muscle fibers, adipocytes, and connective tissue. Furthermore, these elements must exist in the correct 3D distribution. It remains to be determined what level of maturation of muscle fibers and other cells will be necessary to fully recapitulate the organoleptic and nutritional properties of conventional meat, how this process will differ in cocultures of multiple cell types, and whether a different level of maturation will be necessary for structured and unstructured products. It may be feasible to optimize the marbling pattern of CM products by spatially varying the material, stiffness,^[^ ^222^ ^]^ or fat content of the scaffold. For example, fats could be confined to the regions of the scaffold where adipogenic cells are designed to adhere and taken up by adipocytes during the differentiation phase. Consideration should also be given to the organoleptic properties of any remaining scaffold material. For example, scaffolds designed for fish will require recombinant fish collagen, other materials with similarly low melting temperatures, or materials that are degraded and replaced by fish ECM.^[^ ^29^ ^]^

The idea that the nutritional properties of CM could be tuned as desired to achieve certain health outcomes or organoleptic properties^[^ ^110^ ^]^ is worthy of further investigation. In addition to genetic strategies, media optimization offers room for nutritional or flavor enhancement either through the direct addition of beneficial compounds or by adding or removing precursors to helpful or harmful compounds. The scaffold could contribute its own sensory and nutritional qualities, such as by containing microencapsulated flavoring agents or vitamins, contributing to the overall amino acid profile, or increasing fiber content.^[^ ^94^ ^]^ The same technologies needed to recapitulate the naturally occurring fatty acid compositions of certain marine fish, as discussed above, could be applied to freshwater fish, fish lower on the food chain, or terrestrial animals, all of which are naturally lower in EPA, DHA, and related compounds. Future iterations of CM could provide personalized nutrition for humans and pets or even serve as a novel drug delivery system.^[^ ^110^ ^]^

It will be necessary to balance any nutritional enhancements with possible impacts on flavor since fat and its oxidation products can substantially impact the flavor of meat.^[^ ^94^ ^]^ Previous research has shown an increased prevalence of off‐flavors in conventional beef from cattle fed diets high in polyunsaturated fatty acids^[^ ^454^ ^]^ as well as negative correlations between polyunsaturated fatty acid content and tenderness in pork.^[^ ^455^ ^]^ Therefore, it will be important to conduct sensory panels comparing CM prototypes with different levels of nutritional enhancement, both within academic settings to establish general best practices and within private companies to evaluate the impacts of changes to specific products.

CM is an exciting area of scientific innovation that is gaining momentum in both academia and the for‐profit sector. Still, many challenges remain and success is far from inevitable. Because of the unique set of challenges facing CM innovators, interdisciplinary collaboration will be crucial. Experts in tissue engineering, food science, polymer chemistry, mechanical engineering, and many other areas will need to operate at the intersections of their collective knowledge for this technology to reach its full potential.

## Conflict of Interest

The authors declare no conflict of interest.

## Supporting information

Supporting Information
