# Supplementary material for: Scaffolding Biomaterials for 3D Cultivated Meat: Prospects and Challenges
Source: Adv Sci (Weinh). 2021 Nov 16;9(3):2102908. doi: 10.1002/advs.202102908 (PMC8787436; doi:10.1002/advs.202102908)
Supplement: Supplementary file 1 — Supporting Information [file ADVS-9-2102908-s001.pdf]

ADVANCED SCIENCE
Open Access

Supporting Information

for *Adv. Sci.*, DOI: 10.1002/advs.202102908

Scaffolding Biomaterials for 3D Cultivated Meat: Prospects and Challenges

Claire Bomkamp, Stacey C. Skaalure*, Gonçalo F. Fernando, Tom Ben-Arye, Elliot W. Swartz, Elizabeth A. Specht*

Supporting Information

Scaffolding Biomaterials for 3D Cultivated Meat: Prospects and Challenges

Claire Bomkamp, Stacey C. Skaalure*, Gonçalo F. Fernando, Tom Ben-Arye, Elliot W. Swartz, Elizabeth A. Specht*

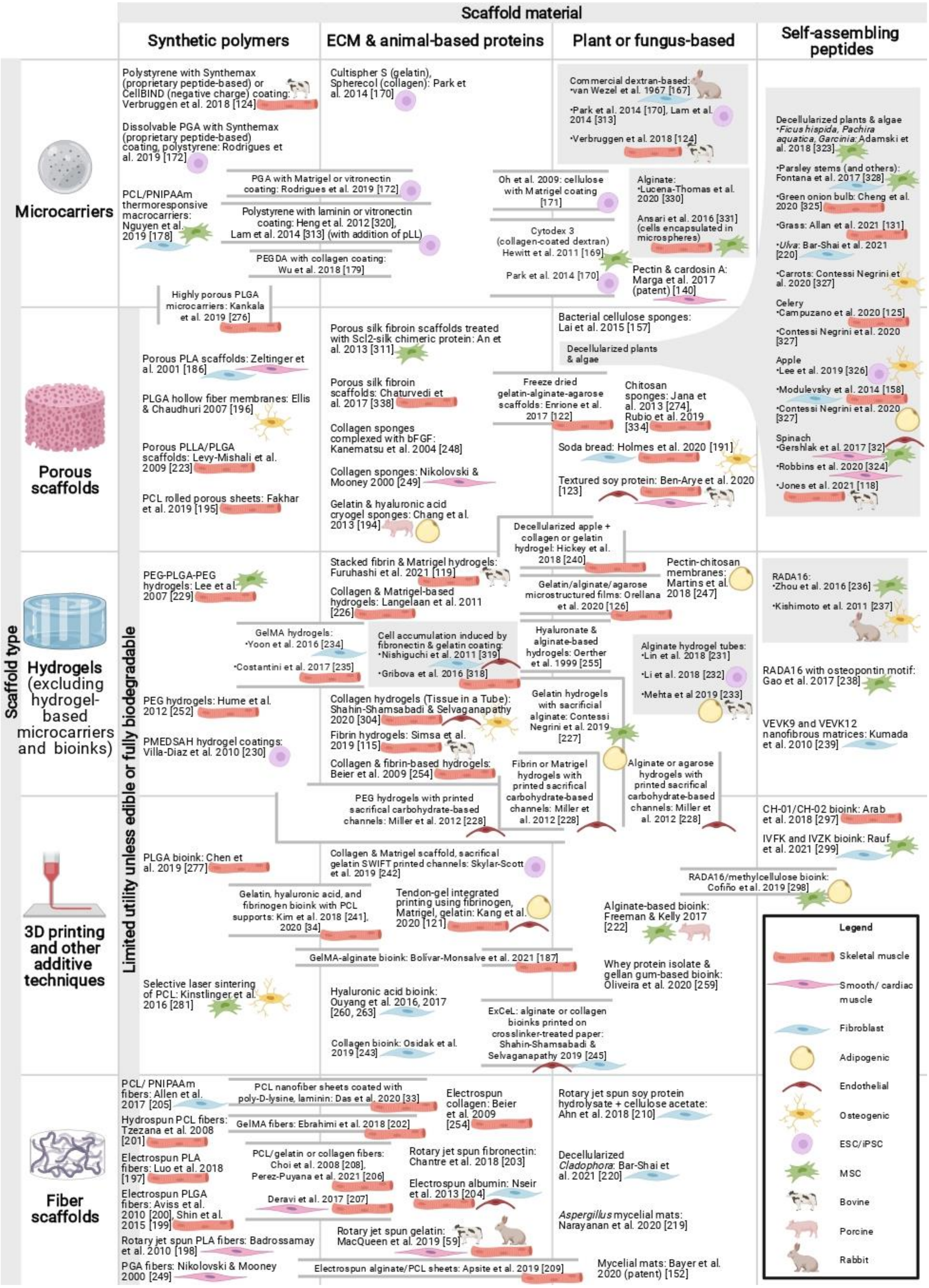

Scaffold material
Synthetic polymers
ECM & animal-based proteins
Plant or fungus-based
Self-assembling peptides
Scaffold type
Microcarriers
Porous scaffolds
Hydrogels (excluding hydrogel-based microcarriers and bioinks)
3D printing and other additive techniques
Fiber scaffolds
Limited utility unless edible or fully biodegradable
Polystyrene with Synthemax (proprietary peptide-based) or CellBIND (negative charge) coating: Verbruggen et al. 2018 [124]
Dissolvable PGA with Synthemax (proprietary peptide-based) coating, polystyrene: Rodrigues et al. 2019 [172]
PCL/PNIPAAm thermoresponsive macrocarriers: Nguyen et al. 2019 [178]
PGA with Matrigel or vitronectin coating: Rodrigues et al. 2019 [172]
Polystyrene with laminin or vitronectin coating: Heng et al. 2012 [320], Lam et al. 2014 [313] (with addition of pLL)
PEGDA with collagen coating: Wu et al. 2018 [179]
Highly porous PLGA microcarriers: Kankala et al. 2019 [276]
Cultispher S (gelatin), Spherecol (collagen): Park et al. 2014 [170]
Commercial dextran-based: •van Wezel et al. 1967 [167]
•Park et al. 2014 [170], Lam et al. 2014 [313]
•Verbruggen et al. 2018 [124]
Oh et al. 2009: cellulose with Matrigel coating [171]
Cytodex 3 (collagen-coated dextran) Hewitt et al. 2011 [169]
Park et al. 2014 [170]
Alginate: •Lucena-Thomas et al. 2020 [330]
Ansari et al. 2016 [331] (cells encapsulated in microspheres)
Pectin & cardosin A: Marga et al. 2017 (patent) [140]
Decellularized plants & algae
•Ficus hispida, Pachira aquatica, Garcinia: Adamski et al. 2018 [323]
•Parsley stems (and others): Fontana et al. 2017 [328]
•Green onion bulb: Cheng et al. 2020 [325]
•Grass: Allan et al. 2021 [131]
•Ulva: Bar-Shai et al. 2021 [220]
•Carrots: Contessi Negrini et al. 2020 [327]
Celery
•Campuzano et al. 2020 [125]
•Contessi Negrini et al. 2020 [327]
Apple
•Lee et al. 2019 [326]
•Modulevsky et al. 2014 [158]
•Contessi Negrini et al. 2020 [327]
Spinach
•Gershlak et al. 2017 [32]
•Robbins et al. 2020 [324]
•Jones et al. 2021 [118]
Porous PLA scaffolds: Zeltinger et al. 2001 [186]
PLGA hollow fiber membranes: Ellis & Chaudhuri 2007 [196]
Porous PLLA/PLGA scaffolds: Levy-Mishali et al. 2009 [223]
PCL rolled porous sheets: Fakhar et al. 2019 [195]
Porous silk fibroin scaffolds treated with Scl2-silk chimeric protein: An et al. 2013 [311]
Porous silk fibroin scaffolds: Chaturvedi et al. 2017 [338]
Collagen sponges complexed with bFGF: Kanematsu et al. 2004 [248]
Collagen sponges: Nikolovski & Mooney 2000 [249]
Gelatin & hyaluronic acid cryogel sponges: Chang et al. 2013 [194]
Bacterial cellulose sponges: Lai et al. 2015 [157]
Decellularized plants & algae
Freeze dried gelatin-alginate-agarose scaffolds: Enrione et al. 2017 [122]
Chitosan sponges: Jana et al. 2013 [274], Rubio et al. 2019 [334]
Soda bread: Holmes et al. 2020 [191]
Textured soy protein: Ben-Arye et al. 2020 [123]
Decellularized apple + collagen or gelatin hydrogel: Hickey et al. 2018 [240]
PEG-PLGA-PEG hydrogels: Lee et al. 2007 [229]
GelMA hydrogels: •Yoon et al. 2016 [234]
•Costantini et al. 2017 [235]
PEG hydrogels: Hume et al. 2012 [252]
PMEDSAH hydrogel coatings: Villa-Diaz et al. 2010 [230]
Stacked fibrin & Matrigel hydrogels: Furuhashi et al. 2021 [119]
Collagen & Matrigel-based hydrogels: Langelaan et al. 2011 [226]
Cell accumulation induced by fibronectin & gelatin coating: •Nishiguchi et al. 2011 [319]
•Gribova et al. 2016 [318]
Collagen hydrogels (Tissue in a Tube): Shahin-Shamsabadi & Selvaganapathy 2020 [304]
Fibrin hydrogels: Simsa et al. 2019 [115]
Collagen & fibrin-based hydrogels: Beier et al. 2009 [254]
Gelatin/alginate/agarose microstructured films: Orellana et al. 2020 [126]
Pectin-chitosan membranes: Martins et al. 2018 [247]
Hyaluronate & alginate-based hydrogels: Oerther et al. 1999 [255]
Alginate hydrogel tubes: •Lin et al. 2018 [231]
•Li et al. 2018 [232]
•Mehta et al 2019 [233]
Gelatin hydrogels with sacrificial alginate: Contessi Negrini et al. 2019 [227]
Fibrin or Matrigel hydrogels with printed sacrifical carbohydrate-based channels: Miller et al. 2012 [228]
Alginate or agarose hydrogels with printed sacrifical carbohydrate-based channels: Miller et al. 2012 [228]
RADA16: •Zhou et al. 2016 [236]
•Kishimoto et al. 2011 [237]
RADA16 with osteopontin motif: Gao et al. 2017 [238]
VEVK9 and VEVK12 nanofibrous matrices: Kumada et al. 2010 [239]
PEG hydrogels with printed sacrifical carbohydrate-based channels: Miller et al. 2012 [228]
PLGA bioink: Chen et al. 2019 [277]
Collagen & Matrigel scaffold, sacrifical gelatin SWIFT printed channels: Skylar-Scott et al. 2019 [242]
Gelatin, hyaluronic acid, and fibrinogen bioink with PCL supports: Kim et al. 2018 [241], 2020 [34]
Tendon-gel integrated printing using fibrinogen, Matrigel, gelatin: Kang et al. 2020 [121]
GelMA-alginate bioink: Bolívar-Monsalve et al. 2021 [187]
Selective laser sintering of PCL: Kinstlinger et al. 2016 [281]
Hyaluronic acid bioink: Ouyang et al. 2016, 2017 [260, 263]
Collagen bioink: Osidak et al. 2019 [243]
ExCeL: alginate or collagen bioinks printed on crosslinker-treated paper: Shahin-Shamsabadi & Selvaganapathy 2019 [245]
Alginate-based bioink: Freeman & Kelly 2017 [222]
Whey protein isolate & gellan gum-based bioink: Oliveira et al. 2020 [259]
CH-01/CH-02 bioink: Arab et al. 2018 [297]
IVFK and IVZK bioink: Rauf et al. 2021 [299]
RADA16/methylcellulose bioink: Cofiño et al. 2019 [298]
PCL/ PNIPAAm fibers: Allen et al. 2017 [205]
Hydrospun PCL fibers: Tzezana et al. 2008 [201]
Electrospun PLA fibers: Luo et al. 2018 [197]
Electrospun PLGA fibers: Aviss et al. 2010 [200], Shin et al. 2015 [199]
Rotary jet spun PLA fibers: Badrossamay et al. 2010 [198]
PGA fibers: Nikolovski & Mooney 2000 [249]
PCL nanofiber sheets coated with poly-D-lysine, laminin: Das et al. 2020 [33]
GelMA fibers: Ebrahimi et al. 2018 [202]
PCL/gelatin or collagen fibers: Choi et al. 2008 [208], Perez-Puyana et al. 2021 [206]
Deravi et al. 2017 [207]
Electrospun collagen: Beier et al. 2009 [254]
Rotary jet spun fibronectin: Chantre et al. 2018 [203]
Electrospun albumin: Nseir et al. 2013 [204]
Rotary jet spun gelatin: MacQueen et al. 2019 [59]
Electrospun alginate/PCL sheets: Apsite et al. 2019 [209]
Rotary jet spun soy protein hydrolysate + cellulose acetate: Ahn et al. 2018 [210]
Decellularized Cladophora: Bar-Shai et al. 2021 [220]
Aspergillus mycelial mats: Narayanan et al. 2020 [219]
Mycelial mats: Bayer et al. 2020 (patent) [152]
Legend
Skeletal muscle
Smooth/ cardiac muscle
Fibroblast
Adipogenic
Endothelial
Osteogenic
ESC/iPSC
MSC
Bovine
Porcine
Rabbit

Figure S1. Summary of scaffold types and materials. Grey outlined items indicate scaffolds belonging to more than one category. Light grey solid boxes are used to indicate groups of similar scaffolds within a category. The "plant- or fungus-based" category also includes molecules commonly produced by these groups that are also sourced from other organisms (e.g., cellulose from bacteria or algae, or chitosan from crustaceans). Scaffolds that have been tested with cell types or cells from species relevant to cultivated meat are indicated with icons to the lower right of the entry.
